# Glass Surfaces Rendered Hydrophobic by Silylation and Sol–Gel Coatings: Optical, Wetting, and Chemical Stability

**DOI:** 10.1021/acsomega.6c02180

**Published:** 2026-09-22

**Authors:** Attila Ábrahám, Kinga Kovács, Lenke Jula-Kócs, Norbert Nagy, Attila Sulyok, Beáta Szolnoki, Dániel Zámbó, Zoltán Hórvölgyi, Emőke Albert

**Affiliations:** † Department of Physical Chemistry and Materials Science, Faculty of Chemical Technology and Biotechnology, Budapest University of Technology and Economics, Műegyetem rkp. 3, H-1111 Budapest, Hungary; ‡ 303347 Institute of Technical Physics and Materials Science, HUN-REN Centre for Energy Research, Konkoly-Thege Miklós út 29-33, H-1121 Budapest, Hungary; § Department of Organic Chemistry and Technology, Faculty of Chemical Technology and Biotechnology, Budapest University of Technology and Economics, Műegyetem rkp. 3, H-1111 Budapest, Hungary

## Abstract

This study presents a comparative evaluation of three strategies for hydrophobizing glass surfaces: direct surface silanization with dimethyldichlorosilane (DMDClSi), the application of a hybrid silica coating (H-SiO_2_), and the use of a hexamethyldisilazane (HMDS)-modified silica coating (P-SiO_2_/HMDS). The glass/H-SiO_2_ and glass/P-SiO_2_/HMDS coatings were prepared by a sol–gel process and deposited onto glass substrates via dip-coating using alcohol-based precursor sols. All surface treatments resulted in hydrophobic surfaces, with advancing water contact angles exceeding 90° and very low contact angle hysteresis (∼1°), indicating uniform and stable water-repellent surfaces. Surface free energy analysis confirmed the predominantly nonpolar character of the modified surfaces, with the lowest values observed for the DMDClSi-treated samples (18 ± 1 mN m^–1^). Both silica-based coatings also increased the light transmittance of the glass substrates compared to the bare glass, achieving maximum transmittance (T_max_) of ≈99.6–99.7% and average transmittance increases of 6.0–6.5% (400–800 nm), together with controlled porosity (34–35%) and uniform thickness values (127 nm). Ellipsometric porosimetry revealed marked differences in pore structures between the silica-based coatings: the glass/H-SiO_2_ coating exhibited a high-surface-area porous network (1124 m^2^ cm^–3^, 38% porosity), whereas the glass/P-SiO_2_/HMDS coating showed higher porosity (48%) with larger mesopores and a lower specific surface area (245 m^2^ cm^–3^), indicating a more open porous structure. The stability of hydrophobic and optical properties was investigated under combined chemical and thermal stress using a three-factor Box–Behnken design. The results identified pH as the main degradation parameter, while temperature and exposure time acted as accelerating factors. Under alkaline–thermal conditions, degradation of Si–CH_3_ groups was identified as the primary mechanism responsible for hydrophobic failure in the glass/H-SiO_2_ system, as confirmed by ATR-FTIR analysis. These results suggest that the high-surface-area pore network of the glass/H-SiO_2_ coating is more susceptible to the loss of Si–CH_3_ functionalities. Wettability measurements demonstrated that the glass/DMDClSi surface exhibited the highest resistance to degradation throughout the investigated design space. Among the silica-based coatings, the glass/P-SiO_2_/HMDS sample showed markedly improved hydrophobic stability compared to glass/H-SiO_2_, retaining water-repellent properties under most conditions and undergoing substantial hydrophilization only under the most severe alkaline–thermal exposure. Light transmittance measurements showed significant optical degradation for glass/H-SiO_2_ (T_max_ ≈ 92–93%) over a wide range of conditions, whereas the glass/P-SiO_2_/HMDS coating maintained high optical stability (T_max_ ≥ 99.1%) across most of the investigated pH-temperature–time combinations, with deterioration observed only under the most severe conditions. Although the DMDClSi treatment provided the highest wettability stability, the glass/P-SiO_2_/HMDS coating combined high optical stability with durable hydrophobicity, and improved resistance to environmental stress.

## Graphical abstract

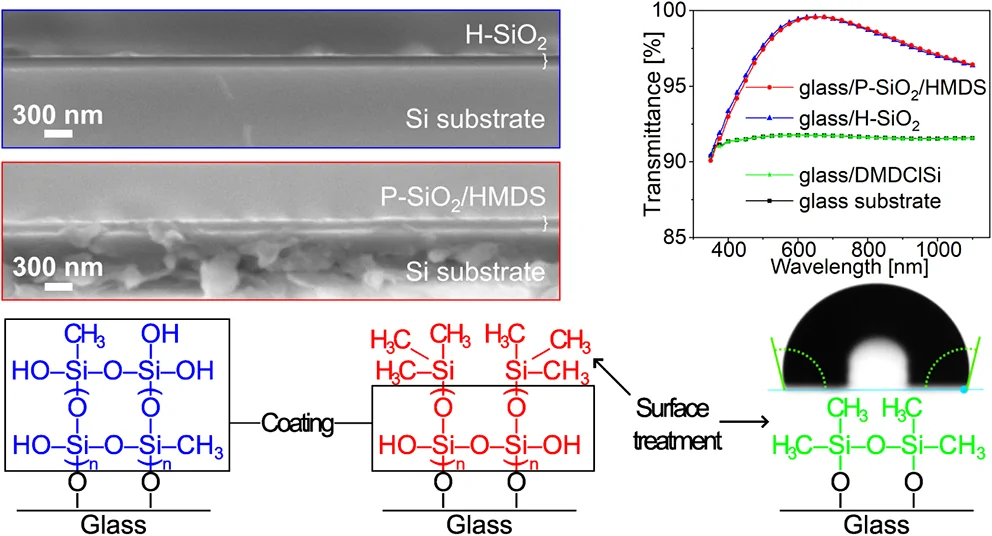

## 1 Introduction

The hydrophobization of glass surfaces has gained significant attention due to its critical role in developing self-cleaning windows, anti-fogging coatings, drag reduction systems, glass displays, and protective cover glasses for electronic devices.^1–3^ However, the practical implementation of treated glass faces substantial challenges, particularly regarding the simultaneous achievement of preserving intrinsic optical transparency and ensuring durable water repellency.^4–6^ The fundamental trade-off between the surface modification necessary for superhydrophobicity and the optical clarity expected from glass represents a critical design challenge that has limited the widespread implementation of these technologies.^7–9^ Recent comprehensive studies have further emphasized that superhydrophobic and multifunctional coatings are increasingly designed not only for water repellency, but also for additional functionalities such as anti-icing, anticorrosion, self-cleaning, and environmental protection performance, highlighting their broad technological relevance and application-driven development trends.^2,3,10–15^ Furthermore, the development of transparent multifunctional interfaces has increasingly relied on advanced hybrid and composite material concepts that integrate surface chemistry and nanoscale structural design within a single coating structure.^2^ Glass substrates can be rendered hydrophobic through various distinct methodologies, primarily either by directly functionalizing the pristine glass surface itself or by applying a secondary coating that inherently possesses or is subsequently modified to exhibit water-repellent properties (e.g., hybrid silica coatings containing nonpolar functional groups integrated into the matrix).^16^ These surface modification strategies typically include silanization-based molecular grafting, which enables the formation of low-surface-energy self-assembled monolayers on hydroxylated glass surfaces;^17^ sol–gel-derived hybrid silica coatings that combine optical transparency with tunable surface functionality;^18,19^ plasma-assisted functionalization, which allows efficient surface activation and subsequent hydrophobic modification;^20^ laser-assisted surface texturing for the generation of controlled micro- and nanoscale roughness;^20^ and nanostructure-assisted surface engineering based on hierarchical sructures that promote superhydrophobic behavior while preserving optical performance.^2,13^ Among these approaches, silanization remains one of the most widely employed methods because organosilanes can form covalently attached self-assembled layers on hydroxylated glass surfaces while simultaneously reducing surface free energy. Early studies demonstrated that chlorosilanes and alkylsilanes can generate densely packed hydrophobic monolayers on silica-based substrates, providing a molecular-level route toward controlled wettability modification and long-term alteration of surface wetting behavior.^12,17^ By carefully optimizing the nanoscale surface structure, thickness, and refractive index of such coatings—particularly silica-based ones—it is possible to develop bifunctional surfaces that simultaneously exhibit antireflective (AR) properties with high light transmittance (>95%) and superhydrophobicity with water contact angles exceeding 150°.^21,22^ Achieving this dual functionality is a delicate process, as it requires minimizing light scattering while maintaining the high apparent contact angles necessary for water repellency.^23,24^ The deposition of a silica coating with an optimized thickness and refractive index on a transparent glass substrate can further enhance its light transmittance.^16^ In addition, functionalizing the glass surface to be hydrophobic enables the development of a bifunctional coating that simultaneously exhibits AR and water-repellent properties.^10,11,21,22,25^ Recent advances in hybrid composite coatings have shown that the incorporation of organic and inorganic building blocks within nanostructured silica matrices enables simultaneous optimization of transparency, wettability, and mechanical stability.^2^ The durability of these bifunctional properties on glass in practical applications presents another significant challenge. Environmental factors such as UV exposure, temperature variations, mechanical stress, and prolonged chemical contact can lead to differential degradation of optical and wetting properties.^5,26,27^ Studies have shown that while the optical properties of treated glass may be partially recoverable through thermal treatment, wetting property changes from surface chemistry modifications are often irreversible.^28^ Long-term stability testing has revealed that coatings can maintain superhydrophobic and antireflective properties on glass for at least one year under outdoor weathering conditions, though mechanical properties may be insufficient for abrasive wear.^24,29^ In this context, recent advances in mechanically robust coating structures have demonstrated that enhancing interfacial adhesion and designing hierarchical structures can significantly improve resistance to abrasion and long-term environmental degradation, which is essential for real-world applications.^30^ Hybrid silica-based materials are particularly promising for developing AR and hydrophobic coatings on glass, as their high chemical compatibility with the substrate allows the formation of bifunctional surfaces in a single preparative step without additional surface modification.^22,31^ Hybrid silica systems combine the excellent optical transparency and chemical stability of inorganic silica networks with the tunable functionality of organic moieties, making them attractive candidates for next-generation multifunctional coatings.^19^ Beyond their structural versatility, hybrid silica systems are increasingly recognized as sustainable and environmentally friendly alternatives to fluorinated coatings, enabling per- and polyfluoroalkyl substance-free surface engineering and aligning with current trends in green chemistry and eco-conscious materials design.^14^ These materials can achieve remarkable optical performance, with transmittance values reaching up to 99.8% while maintaining water contact angles of 90° or higher.^16,24^ The use of intrinsic hydrophobic functionalities, such as Si-CH_n_ units or trimethylsilyl groups, provides more stable water repellency compared to surface-applied fluorination.^31^ Recent studies have further demonstrated that organically modified silica networks can be designed to provide long-term hydrophobicity while maintaining excellent optical properties and improved environmental compatibility.^19^ In addition, polymer–silica hybrid architectures have emerged as a versatile platform for tailoring interfacial properties through molecular-level control of network composition and crosslinking density.^32^ Current research has identified several key design principles for achieving durable transparent superhydrophobic glass: utilizing nanostructures smaller than the visible-wavelength-scattering threshold to minimize light scattering, implementing hierarchical roughness for mechanical resilience, and designing architectures that decouple mechanical support from functional topography to enable independent optimization of each property.^7,8,33,34^ These design strategies are strongly supported by recent advances in multifunctional coating systems, where combined chemical–structural engineering enables simultaneous optimization of optical clarity, mechanical stability, and wetting behavior, reflecting the current trend toward integrated advanced material platforms.^13^ Furthermore, recent studies have highlighted that achieving mechanical robustness remains one of the key limitations of superhydrophobic coatings, and that failure often originates from nanoscale damage accumulation and interfacial delamination under stress.^30^ Advanced coating technologies increasingly employ multiscale composite structures and hybrid material concepts to overcome this limitation while preserving transparency and surface functionality.^2,3^ Recent reports have also demonstrated that combining hierarchical roughness with chemically integrated low-surface-energy functionalities can improve both durability and multifunctionality without relying on environmentally persistent fluorinated compounds.^3,14^ However, significant gaps remain in understanding the long-term stability mechanisms and developing standardized durability testing protocols for these multifunctional glass surfaces.^5,28^

To address these challenges, this study systematically compares three distinct approaches for preparing hydrophobic glass surfaces, including direct silanization and two silica-based coating strategies. While numerous studies have reported individual hydrophobic or antireflective glass treatments, direct comparisons of fundamentally different hydrophobization routes under identical environmental stress conditions are still limited. The novelty of the present work lies in the combined evaluation of surface chemistry, optical performance, wettability, and durability using a unified experimental framework. By combining optical characterization, surface chemical analysis, wettability measurements, and accelerated durability testing across systematically varied chemical (pH 2, 7, and 12), thermal (24.9–59.9 °C), and temporal (2–24 h) conditions, we systematically investigate the interplay between surface chemistry, coating structure, optical performance, and hydrophobic stability. Particular emphasis is placed on evaluating the effectiveness of different hydrophobization strategies in preserving transparency and water repellency under environmental stress. The findings contribute to the rational design of durable, transparent, and multifunctional glass surfaces for optical and protective applications.

## 2 Experimental Section

### 2.1 Materials

All reagents were of analytical grade and used without further purification.

Tetraethoxysilane (TEOS, >99%, Acros Organics), methyl triethoxysilane (MTES, >99%, Sigma-Aldrich), ethanol (EtOH, >99.7%, a.r., Lach-Ner), 2-propanol (2-PrOH, for analysis, Carlo Erba), hydrochloric acid (>36.5%, purum p.a., Fluka), cetyltrimethylammonium bromide (CTAB, >99%, Acros Organics), and ultrapure water (18.2 MΩ·cm, purified with a Millipore Simplicity 185 filtration system) were used during the synthesis of precursor sols.

Ammonium hydroxide (25%, a.r., Lach-Ner) was applied for the post-treatment of the as-deposited coatings, while the Piranha solution was prepared from sulfuric acid (96%, a.r., Carlo Erba) and hydrogen peroxide (30%, a.r., Acros Organics). Dimethyldichlorosilane (DMDClSi, >99%, for analysis, Merck) or hexamethyldisilazane (HMDS, ≥99%, Sigma–Aldrich) were applied as silylating agents. Acetic acid (99.8%, Lach-Ner), phosphoric acid (85%, Lach-Ner), boric acid (a.r., Lach-Ner), hydrochloric acid (HCl, purum p.a., >36.5%, Fluka), sodium hydroxide (NaOH, 98.5%, Acros Organics), and ultrapure water were used during the stability investigations.

Ultrapure water (18.2 MΩ·cm, purified with a Millipore Simplicity 185 filtration system) and diiodomethane (99+%, Acros Organics) were used for wettability measurements.

Microscope glass slides (76 × 26 × 1 mm, Thermo Scientific, Menzel-Gläser) and silicon wafers (Siegert Wafer GmbH, (100), p-type, prime grade) were used as solid substrates.

### 2.2 Synthesis of Precursor Sols

To obtain hybrid silica coatings, a sol from two precursor molecules (TEOS and MTES) was synthesized. First, TEOS was mixed with an aqueous hydrochloric acid solution (0.06 M, pH = 1.3) and ethanol, then the mixture was stirred for 1 h at 60 °C. The molar ratio of TEOS:H_2_O:EtOH was 1:10:7.7. In another beaker, CTAB was dissolved in a mixture of ethanol and MTES and stirred for 30 min at room temperature. The molar ratio of MTES:CTAB:EtOH was 1:0.2:1. The TEOS:MTES molar ratio was 1. After stirring, the two mixtures were poured together and stirred for a further 15 min (400 rpm) at room temperature. The resulting suspension was labeled as H-SiO_2_.^35^

Besides the hybrid silica coating, an alternative silica sol containing Pluronic PE 10500 was synthesized and used for the preparation of a second type of silica coating (P-SiO_2_). This silica sol was synthesized by the acid-catalyzed controlled hydrolysis of TEOS. The molar ratio of the components was TEOS:2-propanol:HCl:H_2_O:PE10500 = 1:14.49:0.01:5.61:0.01. In the first step, the Pluronic PE 10500 surfactant and a 0.1 M hydrochloric acid solution were added to the alcoholic medium, and it was stirred (400 rpm) until the complete dissolution of the surfactant (ca. 30 min) at 25 °C. TEOS was added to this solution, and the mixture was stirred for further 30 min (400 rpm).^36^

### 2.3 Preparation of the Samples

The overall sample preparation strategy was designed to obtain two coating types on glass substrates (glass/H-SiO_2_ and glass/P-SiO_2_/HMDS) with closely similar optical and wetting properties. Therefore, on the basis of our previous experience and preliminary experimental results, the specific sols, processing parameters, environmental conditions, and post-treatment steps described in the following subsections were optimally selected and applied.

#### 2.3.1 Preparation of the Sol–Gel Coatings

Microscope glass slides and silicon wafers were used as solid substrates for layer deposition. Prior to film deposition, glass substrates were washed with an aqueous detergent solution (1 wt %), rinsed with ultrapure water, and neutralized with a dilute sulfuric acid solution (10 wt %). After that, they were wiped with 2-propanol-impregnated cotton, then rinsed with 2-propanol and ultrapure water. Silicon substrates were cleaned with 2-propanol and ultrapure water. Then the substrates were dried at 70 °C in a drying oven (Memmert UNB 400).

Prior to layer deposition, the silicon substrates were treated with O_2_ plasma (10 min, 0.4 mbar, 300 W) using a radiofrequency-generated low-pressure plasma system (SmartPlasma 10, Plasma Technology GmbH) coupled with an oxygen concentrator (DeVilbiss Healthcare 525 KS) in order to enhance surface adhesion and wettability toward the alcoholic precursor solutions.

Sol–gel coatings on glass and silicon substrates were prepared from the precursor sols by the dip-coating method (dip coater, Plósz Mérnökiroda Kft., Hungary) at room temperature. Cleaned and dried substrates were immersed in the precursor sol and pulled out at a constant speed of 5 cm min^–1^ (H-SiO_2_ sol) or 1.5 cm min^–1^ (P-SiO_2_ sol). The deposited H-SiO_2_ coatings were annealed in a muffle furnace (Nabertherm B170) at 400 °C for 2 h with a heating rate of 13.3 °C min^–1^. In the case of P-SiO_2_ coatings, colloidal aging was applied in order to strengthen their structure. The as-deposited coatings were placed in a sealed desiccator above 250 mL of a 2 M aqueous ammonium hydroxide solution and exposed to aqueous ammonia vapor for 2 h at ambient temperature (22–26 °C). The volume of the desiccator was 11 L. The treated samples were annealed in a muffle furnace (Nabertherm B170) at 480 °C for 1 h with a heating rate of 15.0 °C min^–1^. Finally, the samples were immersed in ultrapure water for 30 min and dried at 70 °C.

#### 2.3.2 Silylation Methods

Silylated glass and silicon substrates were prepared by using a 1% v/v% DMDClSi solution in *n*-hexane. Prior to silylation, glass substrates were immersed in Piranha solution (a 3:1 mixture of H_2_SO_4_ and H_2_O_2_) for 5 min and then rinsed with ultrapure water. Before hydrophobization, the silicon substrates were subjected to plasma treatment (O_2_ plasma, 10 min, 0.4 mbar, 300 W). Subsequently, both glass and silicon substrates were immersed in the silylating agent solution for 1 h. After the reaction, samples were rinsed with *n*-hexane which was followed by heat treatment at 150 °C for 30 min.

Silylated silica and glass (for comparison) surfaces were obtained by HMDS vapor treatment. Prior to silylation, the porous samples were kept in 0.01 M HCl solution for 5 min in order to induce the hydrolysis of alkoxy groups still present on the surface. The silica and glass (for comparison) samples were kept in a desiccator (ca. 1 L) containing 0.5 mL of HMDS reagent for 6 h. Then the samples were heat-treated at 350 °C, for 1 h, with a heating rate of 5 °C/min.

### 2.4 Characterization Methods

#### 2.4.1 UV–Visible Spectroscopy (UV–Vis)

The optical properties of the samples on glass substrates were measured by UV–Vis spectroscopy (Analytik Jena, Specord 200). Transmittance spectra of the uncoated glass substrates and the coated samples were measured in the wavelength range of 350–1100 nm with a 1 nm resolution and a 5 nm s^–1^ scanning speed at normal incidence.

From the transmittance spectra, the maximum light transmittance (T_max_) and the average transmittance increase (ATI) in the visible light range (400–800 nm) were determined. The calculation of ATI is described by eq 1.

$$\mathrm{ATI}=\frac{A_{\mathrm{sample}}-A_{\mathrm{substrate}}}{800-400}$$ 1

where A_sample_ and A_substrate_ stand for the integrated area in the investigated wavelength range of the transmittance spectra measured on the coated sample and the uncoated substrate, respectively.^37,38^ Effective refractive index and layer thickness values were determined from the transmittance spectra using a thin-layer optical model, assuming a perpendicular angle of incidence and identical homogeneous monolayers of the same material on both sides of the transparent substrate (Hild model).^39,40^ The fitting procedure was performed using the Levenberg–Marquardt algorithm. The porosity of the coatings was calculated by the Lorentz–Lorenz equation.^41^

#### 2.4.2 Ellipsometric Porosimetry (EP)

Ellipsometric porosimetry was used to determine the open porosity, pore size, and pore size distribution of the glass/H-SiO_2_ and glass/P-SiO_2_/HMDS samples. Measurements were carried out using a Semilab Sopra EP-12 ellipsometric porosimeter. EP is a well-established technique for the characterization of porous thin films, providing information on open porosity, specific surface area, pore size, and mechanical strength. The method combines vapor adsorption with spectroscopic ellipsometry, enabling the adsorption process to be monitored stepwise. During the measurements, vapor of the adsorptive material condenses within the pore network, resulting in a change in the effective refractive index. Analysis of the adsorption–desorption isotherms allows the determination of sample porosity, while the pore size distribution can be calculated using the modified Kelvin equation for mesopores and the Dubinin–Radushkevich model for micropores. The SiO_2_ coatings were characterized using 2-PrOH as the adsorptive material. Prior to the measurements, the samples were heated at 175 °C for 15 min and then vacuumed. The 2-propanol adsorption–desorption isotherms were recorded at 24 °C. In addition, EP enables the determination of the optical properties of the samples at zero relative pressure of the adsorptive material, similar to conventional spectroscopic ellipsometry. Measurements were performed at an angle of incidence of 60° over a wavelength range of 248.5–971.5 nm. The coating thickness and effective refractive index (at 632.8 nm) were determined using the Cauchy dispersion model. The specific surface area values were calculated according to the BET method, assuming a molecular cross-sectional area of 0.388 nm^2^ for 2-PrOH.^42^

#### 2.4.3 Wettability Measurements

Wettability properties of the samples were studied by the determination of advancing (Θ_A_) and receding (Θ_R_) water contact angles (CA). The contact angles were measured with a drop shape analyser (DSA30, KRÜSS GmbH) using the sessile drop method and the drop-build-up technique. Advancing contact angles were determined using a drop volume of 10 μL, and in the case of receding angles, a diminished volume of 5 μL (removing 5 μL from the 10 μL drop) was used. Measurements were performed in a closed chamber with a relative humidity greater than 95% on three parallel samples at 25.0 °C. The wetting liquid was ultrapure water. Advancing and receding values of water contact angles were determined from four droplets on each sample. The contact angle hysteresis (H) was calculated as the difference between the advancing (Θ_A_) and receding (Θ_R_) contact angles according to eq 2:

2 $$H=\Theta_{A}-\Theta_{R}$$

To evaluate the interfacial wetting energetics, the work of adhesion (W_adh_) was determined via the classical Young–Dupré equation^43^ (eq 3):

3 $$W_{\mathrm{adh}}=\gamma_{LV}\left(1+\cos\Theta_{\mathrm{Young}}\right)$$

where γ_LV_ represents the liquid–vapor surface tension (for water at 25 °C, γ_LV_ = 72.8 mN m^–1^),^44^ and θ_Young_ is the theoretical Young (equilibrium) contact angle. The equilibrium contact angle was calculated using the Wolfram–Faust approximation^45^ (eq 4):

4 $$\cos\theta_{\mathrm{Young}}\approx \frac{\cos\theta_{A}+\cos\theta_{R}}{2}$$

The surface free energy (SFE) of the samples was determined from advancing contact angles measured at 25 °C using ultrapure water and diiodomethane as probe liquids. Droplets of 10 μL volume were deposited onto the sample surfaces, and their profiles were analyzed using Krüss Advance software. For determination of the surface free energy, the surface tension and its polar and dispersive components for water and diiodomethane at 25 °C were used. The corresponding values were as follows: water ( γ_LV_= 72.8 mN m^–1^, polar component = 51.0 mN m^–1^, dispersive component = 21.8 mN m^–1^) and diiodomethane ( γ_LV_= 50.8 mN m^–1^, polar component = 0 mN m^–1^, dispersive component = 50.8 mN m^–1^).^46,47^ Contact angle values were obtained by ellipse fitting, and the surface free energy was calculated according to the Owens–Wendt–Rabel–Kaelble (OWRK) two-liquid model.^46^

#### 2.4.4 X-ray Photoelectron Spectroscopy (XPS)

To characterize the surface coating, the elemental composition in the surface range was examined by X-ray photoelectron spectroscopy (XPS) using an Al anode. Spectra were obtained by a special cylindrical mirror analyzer with retarding field (type DESA 150, Staib Instruments Ltd.) that provided a constant energy resolution of 1.5 eV. After taking a spectrum from the freshly prepared surface, further spectra were recorded after applying a gentle low-energy ion bombardment (Ar, 1 keV, 78° angle of incidence) to remove possible surface contamination from the environment. The chemically different subcomponents of each XPS lines were determined with a peak fitting algorithm with Shirley background subtraction and Gaussian/Lorentzian fitting.

#### 2.4.5 Abrasion Resistance Test

The abrasion resistance was evaluated using a Coating Hardness Kit (Summers Optical, a division of EMS Acquisition Corp.) in accordance with ISO 9211-4:2012.^48^ A stack of 32 layers of cotton cheesecloth (MIL CCC-C-440) was prepared and used as the abrasive medium. The head of the aluminum abrasion tester was covered with the cheesecloth pad and loaded with a force of 5 N. The tester was then manually moved across the surfaces of the glass/H-SiO_2_ and glass/P-SiO_2_/HMDS samples. A total of 100 rubbing strokes were applied to each sample, consisting of 50 forward and 50 backward passes. After the abrasion test, the sample surfaces were visually inspected and compared to evaluate abrasion-induced damage and abrasion resistance of the coatings.

#### 2.4.6 Soaking Experiments

To evaluate the environmental durability of the samples, soaking experiments were conducted according to a three-factor, three-level Box–Behnken experimental design.^49,50^ The three factors were temperature of the soaking media, soaking time, and the pH of the soaking media. The experimental ranges and levels of the independent variables considered in this study are presented in Table 1. The pH was kept at constant values with Britton-Robinson buffer solutions.^51^

**Table 1:** Experimental Range and Levels of Independent Variables in the Soaking Experiments

	**Range and levels**		
**Variables**	**Low level (−1)**	**Center level (0)**	**High level (+1)**
**Temperature of the soaking media [°C]**	24.9	42.4	59.9
**Soaking time [h]**	2.0	13.0	24.0
**pH of the media [−]**	2.0	7.0	12.0

After soaking, the samples were rinsed with ultrapure water and dried at room temperature, then the changes in the samples’ wettability and optical properties were evaluated with contact angle measurements and UV–Vis spectroscopy. The obtained data were evaluated with the following quadratic model^52^ (eq 5):

5 $$Y=\beta_{0}+\overset{k}{\underset{i=1}{\sum }}\beta_{i}x_{i}+\overset{k}{\underset{i=1}{\sum }}\beta_{\mathrm{ii}}x_{i}^{2}+\overset{k}{\underset{i=1}{\sum }}\overset{k}{\underset{j=1}{\sum }}\beta_{\mathrm{ij}}x_{i}x_{j}+\varepsilon$$

where Y is the dependent variable, k is the number of the variables, i and j are the index numbers for the variables, β_0_ is the free or offset term called the intercept term, x_i_ is the coded independent variable, β_i_ is the first-order (linear) main effect, β_ii_ is the quadratic (squared) effect, β_ij_ is the interaction effect, and ε is the random error term accounting for discrepancies or uncertainties between predicted and measured values.

#### 2.4.7 Field-Emission Scanning Electron Microscopy (FE-SEM)

Samples on silicon substrates were investigated by field-emission scanning electron microscopy (FE-SEM). Top-view and cross-sectional FE-SEM images were taken by using a Zeiss LEO 1540-XB field-emission scanning electron microscope operated at an accelerating voltage of 5.0 kV.

#### 2.4.8 Atomic Force Microscopy (AFM)

Surface morphology of the samples on glass substrates was analyzed with an AIST-NT SmartSPM 1000 Atomic Force Microscope (AFM) setup operated in tapping mode. The measured AFM data were processed using Gwyddion software.^53^

#### 2.4.9 Attenuated Total Reflectance Fourier Transform Infrared Spectroscopy (ATR-FTIR) Measurements

ATR-FTIR measurements were carried out on the as-prepared and soaked (pH 12, 59.9 °C,13 h) glass/H-SiO_2_ samples, as well as on an untreated glass substrate (for comparison). The FTIR spectra were taken in the wavenumber range of 4000–600 cm^–1^ with an FTIR spectrometer (InfraRed Bruker Tensor 37) equipped with a deuterated triglycine sulfate detector with a resolution of 4 cm^–1^.

## 3 Results and Discussion

### 3.1 Optical Properties of the As-Prepared Samples: UV–Vis Spectroscopy

The light transmittance of the glass substrate, the glass substrate hydrophobized with DMDClSi (glass/DMDClSi), the hybrid silica coating on the glass substrate (glass/H-SiO_2_), and the silica coating on the glass substrate hydrophobized with HMDS (glass/P-SiO_2_/HMDS) was investigated by UV–Vis spectroscopy (Figure 1). Chemical surface modification with DMDClSi did not affect the light transmittance of the glass substrate, as the transmittance curves of the glass/DMDClSi sample and the bare glass substrate overlap in the whole studied wavelength range. Both the glass/H-SiO_2_ and glass/P-SiO_2_ samples show a considerable increase in light transmittance compared to the bare glass substrate, and their transmittance spectra are practically identical within experimental error (Figure 1). They exhibit T_max_ values of 99.60 ± 0.07% and 99.68 ± 0.05%, and average transmittance increases of 6.5 ± 0.1% and 6.0 ± 0.2% in the 400–800 nm wavelength range, respectively (Table 2). The slightly lower ATI of the glass/P-SiO_2_/HMDS sample corresponds to the minor difference in effective refractive index between the two samples. Thickness and effective refractive index values of the H-SiO_2_ and P-SiO_2_/HMDS coatings were determined based on thin-layer optical models by applying fitting procedures^39,40^ (Table 2). Porosity values were calculated by the Lorentz–Lorenz^41^ formula in view of the effective refractive indices of the silica coatings and the refractive indices of bulk SiO_2_ (n_bulk_ = 1.460) and air (n_air_ = 1) (Table 2). Effective refractive indices of 1.284 ± 0.004 and 1.289 ± 0.003 (corresponding to porosities of 35% and 34%, respectively), and an identical layer thickness of 127 nm were obtained for the H-SiO_2_ and P-SiO_2_/HMDS coatings (Table 2).

**Figure 1:**
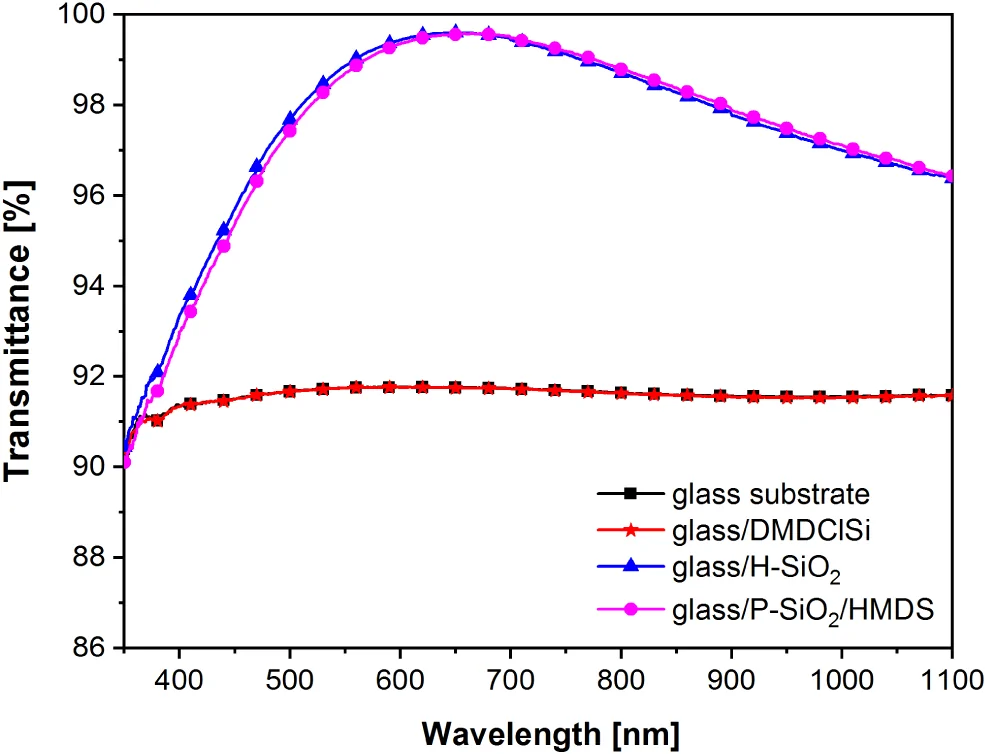
Representative transmittance spectra of the glass substrate, the glass substrate hydrophobized with DMDClSi (glass/DMDClSi), the hybrid silica coating (glass/H-SiO_2_), and the silica coating hydrophobized with HMDS (glass/P-SiO_2_/HMDS) on glass substrates.

**Table 2:** Maximum Transmittance (T_max_), Average Transmittance Increase (ATI, in the 400–800 nmWavelength Range, Compared to Their Bare Glass Substrates), Effective Refractive Index (n), Layer Thickness (d), and Porosity (P, Lorentz–Lorenz) Values of the Glass/H-SiO_2_ and Glass/P-SiO_2_/HMDS Samples

**Sample**	**T_max_ [%]** **(λ** at **T_max_)**	**ATI [%]** **(400–800 nm)**	**n [−]**(**632.8 nm)**	**d [nm]**	**P [%]** **(Lorentz–Lorenz)**
**Glass/H-SiO_2_**	99.60 ± 0.07 (656 ± 6 nm)	6.5 ± 0.1	1.284 ± 0.004	127 ± 1	35 ± 1
**Glass/P-SiO_2_/HMDS**	99.68 ± 0.05 (682 ± 5 nm)	6.0 ± 0.2	1.289 ± 0.003	127 ± 2	34 ± 1

### 3.2 Optical Properties of the As-Prepared Samples: Ellipsometric Porosimetry

The pore structure of the glass/H-SiO_2_ and glass/P-SiO_2_/HMDS coatings was characterized by ellipsometric porosimetry using 2-propanol as the adsorptive at 24 °C. The glass/H-SiO_2_ sample exhibits a steep adsorption step in the relative pressure range of p/p_0_ ≈ 0.40–0.55 with only minor hysteresis between the adsorption and desorption branches (Figure 2a), indicating a relatively homogeneous mesoporous network with a narrow pore size distribution. The limited extent of hysteresis suggests efficient pore emptying and the absence of pronounced pore-network effects. In contrast, the glass/P-SiO_2_/HMDS sample (Figure 2b) displays a type IV isotherm with a pronounced H2-type hysteresis loop according to the IUPAC classification. Capillary condensation occurs at substantially higher relative pressures (p/p_0_ ≈ 0.80–0.95), while the broad hysteresis loop indicates the presence of interconnected mesopores, pore constrictions, and significant pore-network effects associated with a structurally heterogeneous porous structure.^54,55^

**Figure 2:**
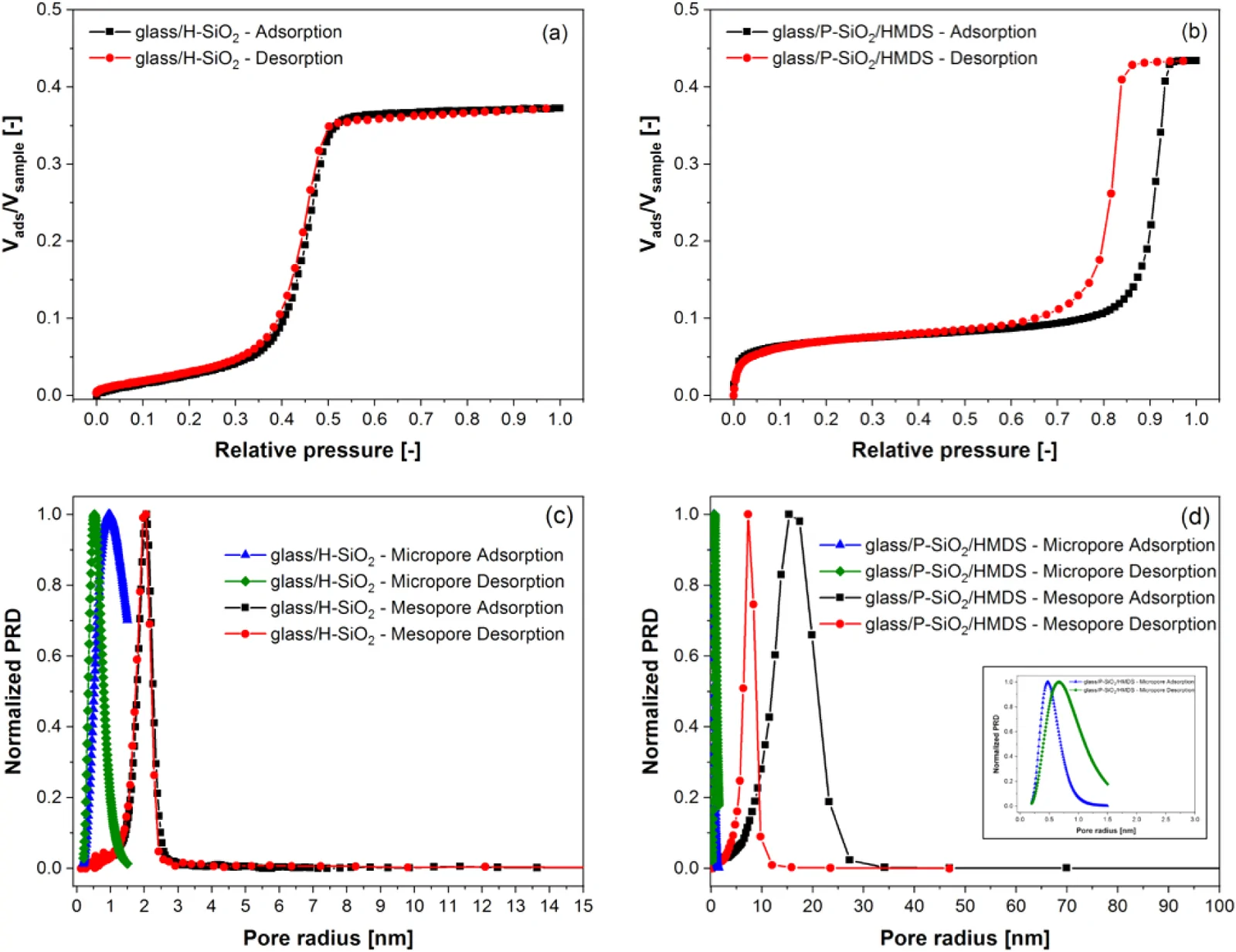
2-Propanol adsorption–desorption isotherms taken at 24 °C (a,b) and the corresponding normalized pore radius distributions (c, d) for the glass/H-SiO_2_ (a, c) and glass/P-SiO_2_/HMDS (b, d) samples, determined by ellipsometric porosimetry. Inset in (d): a magnified view of the microporous pore radius distribution.

The normalized pore radius distributions shown in Figure 2c and d were derived from the adsorption isotherms (Figure 2a and b) by applying the Dubinin–Radushkevich model in the micropore range and the modified Kelvin equation in the mesopore range. In addition, the effective refractive index (at 632.8 nm) and coating thickness values were determined by spectroscopic ellipsometry at zero relative pressure using the Cauchy dispersion model (Table 3). The total porosity was subsequently calculated from the effective refractive index values using the Lorentz–Lorenz equation (Table 3). The ellipsometric porosimetry results reveal substantial differences in the pore structure of the glass/H-SiO_2_ and glass/P-SiO_2_/HMDS coatings (Table 3). The glass/P-SiO_2_/HMDS sample exhibits a lower effective refractive index (n = 1.225) than the glass/H-SiO_2_ coating (n = 1.259), indicating a higher pore fraction within the coating. This is consistent with the total porosity values calculated using the Lorentz–Lorenz equation, which are 38% for glass/H-SiO_2_ and 48% for glass/P-SiO_2_/HMDS. The corresponding open porosity values are 37% and 43% for the glass/H-SiO_2_ and glass/P-SiO_2_/HMDS samples, respectively (Table 3). These results suggest differences in pore accessibility and connectivity within the two coating systems. This interpretation is supported by the mesopore radii obtained from the adsorption and desorption branches of the isotherms. While the glass/H-SiO_2_ sample exhibits identical adsorption and desorption mesopore radii (2.1 nm), indicating a relatively uniform mesoporous network, the glass/P-SiO_2_/HMDS coating shows a pronounced difference between the adsorption and desorption mesopore radii (15.4 nm and 7.3 nm, respectively). Such behavior suggests the presence of pore-network effects and possible pore constrictions, which may limit access to part of the pore volume. The pore size distributions further highlight the structural differences between the two coatings. The glass/H-SiO_2_ sample is characterized by micropore radii of 1.0 nm (adsorption) and 0.5 nm (desorption), together with a relatively narrow mesopore size distribution centered at approximately 2.1 nm. In contrast, the glass/P-SiO_2_/HMDS coating exhibits micropore radii of 0.5 nm (adsorption) and 0.7 nm (desorption), while the mesoporous pore-size range is shifted toward substantially larger pore sizes. These results demonstrate that the HMDS-modified coating possesses a broader pore structure with significantly larger mesopores than the hybrid silica coating. The specific surface area also differs markedly between the two samples. The glass/H-SiO_2_ coating exhibits a high BET specific surface area of 1124 m^2^ cm^–3^, whereas 245 m^2^ cm^–3^ was obtained for the glass/P-SiO_2_/HMDS coating. This decrease is consistent with the substantial increase in characteristic pore size, as larger pores provide a lower internal surface area per unit volume than smaller pores. Consequently, although the HMDS-modified coating possesses a higher overall porosity, its accessible internal surface area is considerably lower than that of the hybrid silica coating. Overall, the ellipsometric porosimetry results demonstrate that the glass/H-SiO_2_ coating consists of a relatively homogeneous micro- and mesoporous network with high specific surface area, whereas the glass/P-SiO_2_/HMDS coating is characterized by higher total porosity, substantially larger mesopores, and more complex pore accessibility. These structural differences are reflected in the refractive index, porosity, specific surface area, and pore size distribution values determined for the two coating systems (Table 3). Although minor differences exist between the optical and porosity-related parameters obtained from ellipsometric porosimetry and UV–Vis spectroscopy, these deviations mainly arise from the different measurement principles and evaluation methodologies of the two techniques, while both methods lead to the same overall conclusions regarding the structure–property relationships of the investigated coatings.

**Table 3:** Effective Refractive Index (n, 632.8 nm), Thickness (d), Total Porosity (Total P, Lorentz–Lorenz), and Open Porosity (Open P), Specific Surface Area (BET), and Pore Radius (r_ads_, r_des_) Values for the Microporous (Dubinin–Radushkevich) and Mesoporous (Modified Kelvin) Ranges of the Glass/H-SiO_2_ and Glass/P-SiO_2_/HMDS Samples, Determined by Ellipsometric Porosimetry

**Sample**	**n [−] (632.8 nm)**	**d [nm]**	**Total P [%]**	**Open P [%]**	**S_BET_** **[m^2^** **/cm^3^**]	**r_ads_ micropores [nm]**	**r_des_ micropores [nm]**	**r_ads_ mesopores [nm]**	**r_des_ mesopores [nm]**
**Glass/H-SiO_2_**	1.259	134	38	37	1124	1.0	0.5	2.1	2.1
**Glass/P-SiO_2_/HMDS**	1.225	108	48	43	245	0.5	0.7	15.4	7.3

### 3.3 Wettability and Surface Free Energy of the As-Prepared Samples

Wettability of the samples was studied by the sessile-drop method with ultrapure water. The advancing contact angle (θ_A_), receding contact angle (θ_R_), and contact angle (CA) hysteresis (H) values determined for the glass substrate hydrophobized with DMDClSi (glass/DMDClSi), the hybrid silica sample (glass/H-SiO_2_) and the silica sample hydrophobized with HMDS (glass/P-SiO_2_/HMDS) are presented in Table 4. All investigated surfaces exhibit hydrophobic behavior, with measured advancing and receding contact angles exceeding 90°. The hydrophobized glass (glass/DMDClSi) sample shows the highest advancing contact angle (101 ± 1°), indicating the highest hydrophobicity among the studied samples. The glass/H-SiO_2_ and glass/P-SiO_2_/HMDS coatings show slightly lower advancing contact angles of 95 ± 1° and 93 ± 1°, respectively, while remaining within the hydrophobic range (Table 4). The contact angle hysteresis is 1° in all cases, indicating a high degree of chemical and morphological homogeneity of the surfaces. The equilibrium (Young) contact angles calculated for the investigated samples are in close agreement with the advancing contact angle values, consistent with the very low contact angle hysteresis (Table 4). The Young contact angle decreases in the order glass/DMDClSi (101 ± 1°) > glass/H-SiO_2_ (95 ± 1°) > glass/P-SiO_2_/HMDS (93 ± 1°), indicating a gradual increase in the affinity of the surfaces toward water. This trend is also reflected in the work of adhesion values calculated according to the Young–Dupré equation (Table 4). The hydrophobized glass substrate (glass/DMDClSi) exhibits the lowest work of adhesion (60 ± 1 mN m^–1^), whereas the glass/H-SiO_2_ and glass/P-SiO_2_/HMDS samples show higher values of 67 ± 1 mN m^–1^ and 70 ± 1 mN m^–1^, respectively. The increase in adhesion work corresponds to the decrease in Young contact angle and suggests stronger interfacial interactions between water and the silica-based coatings compared to the DMDClSi-modified glass surface. Nevertheless, the relatively low work of adhesion values and Young contact angles above 90° confirm the hydrophobic character of all investigated surfaces, despite the slightly enhanced wettability observed for the silica-containing coatings.

**Table 4:** Advancing (θ_A_) Contact Angle, Receding (θ_R_) Contact Angle, Contact Angle Hysteresis (H), Equilibrium (Young) Contact Angle (θ_Young_), and Work of Adhesion (W_adh_) Values Determined with Ultrapure Water for the Glass Substrate Hydrophobized with DMDClSi (Glass/DMDClSi), the Hybrid Silica Coating (Glass/H-SiO_2_), and the Silica Coating Hydrophobized with HMDS (Glass/P-SiO_2_/HMDS) on Glass Substrates

**Sample**	**Θ_A_ [°]**	**Θ_R_ [°]**	**H [°]**	**Θ_Young_ [°]**	**W_adh_** **[mN m** ^–1^
**Glass/DMDClSi**	101 ± 1	100 ± 1	1 ± 1	101 ± 1	60 ± 1
**Glass/H-SiO_2_**	95 ± 1	94 ± 1	1 ± 1	95 ± 1	67 ± 1
**Glass/P-SiO_2_/HMDS**	93 ± 1	92 ± 1	1 ± 1	93 ± 1	70 ± 1

The surface free energy (SFE) of the glass/DMDClSi and glass/H-SiO_2_ samples was determined from advancing contact angle measurements performed using ultrapure water and diiodomethane as probe liquids, at 25 °C. The surface free energy was calculated according to the Owens–Wendt–Rabel–Kaelble (OWRK) two-liquid model. The contact angles measured on the surfaces of the investigated samples using ultrapure water and diiodomethane, along with the corresponding surface free energy values calculated from these measurements, including the dispersive and polar components, are summarized in Table 5. The SFE results are fully consistent with the previously discussed wettability trends and provide further insight into the interfacial characteristics of the investigated surfaces. The lowest total SFE is observed for the glass/DMDClSi sample (18 ± 1 mN m^–1^), in agreement with its highest water contact angle and lowest work of adhesion (Tables 4 and 5). This confirms that the DMDClSi modification yields a surface with strongly reduced surface free energy. The SFE is dominated by its dispersive component (16 ± 1 mN m^–1^), while the polar contribution remains negligible (2 ± 1 mN m^–1^), indicating the absence of significant polar functionalities at the outermost surface. For the glass/H-SiO_2_ sample, the total surface free energy increases to 26 ± 1 mN m^–1^, in agreement with the previously observed decrease in contact angle and increase in adhesion work relative to the glass/DMDClSi sample (Tables 4 and 5). This increase is primarily attributed to the higher dispersive component (24 ± 1 mN m^–1^), whereas the polar component remains low (2 ± 1 mN m^–1^), similar to the DMDClSi-modified surface. The comparison between the two systems indicates that the surface modification primarily affects the dispersive component of the surface free energy, while the polar contribution remains essentially unchanged. The increase in SFE from the DMDClSi-modified surface to the hybrid silica coating correlates with the decrease in Young’s contact angle and the increase in adhesion work discussed above. Overall, the SFE results confirm that all investigated surfaces are in the hydrophobic range, with the observed differences in wettability driven by minor variations in surface energy components.

**Table 5:** Contact Angles Measured with Ultrapure Water and Diiodomethane on the Surfaces of Glass/DMDClSi and Glass/H-SiO_2_ Samples, Together with the Corresponding Surface Free Energy Values and Their Polar and Dispersive Components at 25 °C, Determined Using the Two-Liquid Owens–Wendt–Rabel–Kaelble (OWRK) Model

**Sample**	**Water contact angle [°]**	**Diiodo-methane contact angle [°]**	**Surface free energy** **[mN m^–1^]**	**Polar component of surface free energy**[**mN m^–1^**]	**Dispersive component of surface free energy** **[mN m^–1^]**
**Glass/DMDClSi**	102 ± 1	83 ± 2	18 ± 1	2 ± 1	16 ± 1
**Glass/H-SiO_2_**	95 ± 1	67 ± 1	26 ± 1	2 ± 1	24 ± 1

### 3.4 Surface Chemical Composition by X-ray Photoelectron Spectroscopy

The surface chemical composition of the glass substrate hydrophobized with DMDClSi (glass/DMDClSi), the glass substrate hydrophobized with HMDS (glass/HMDS, for comparison), and the silica coating on glass substrate hydrophobized with HMDS (glass/P-SiO_2_/HMDS) was determined by XPS. The elemental composition of the surfaces of the investigated samples could be calculated from peak intensities assuming a homogeneous distribution of elements using the elemental weight factors shown in Table 6.^56^ This provides a general overview of the surface elemental composition. Since we definitely know that the composition is not homogeneous (instead layered) within the information depth of XPS, a more refined evaluation was applied to determine the layer thickness above the glass substrate. A layer-on-substrate model was elaborated to describe the observed experimental peak intensities. The model included the stoichiometry of the deposited layers as well as the mean free paths of photoelectrons.^57^ Achieving the simulation of the experimental spectra, the model provided the average thickness of the coatings. For the DMDClSi-modified glass surface, a hydrophobic layer with a thickness of 8.6 ± 0.1 Å was formed, whereas significantly thinner layers were obtained on the HMDS-modified glass (glass/HMDS) and (glass/P-SiO_2_/HMDS) surfaces, with thicknesses of 3.2 ± 0.3 Å and 2.9 ± 0.7 Å, respectively. This indicates that the type of silylating agent plays a decisive role in determining the thickness of the surface hydrophobic layer, whereas under the experimental conditions applied, the substrate type does not significantly affect the layer thickness.

**Table 6:** XPS Observed Average Chemical Composition at the Surface of the Glass Substrate Hydrophobized with DMDClSi (Glass/DMDClSi), the Glass Substrate Hydrophobized with HMDS (Glass/HMDS), and the Silica Coating Hydrophobized with HMDS (Glass/P-SiO_2_/HMDS)

	**Elemental composition [at %]**					
**Sample**	**C**	**O**	**Si**	**Mg**	**Na**	**Cl**
**Glass/DMDClSi**	14.7 ± 0.1	51.0 ± 0.1	29.4 ± 0.2	1.0 ± 0.1	3.6 ± 0.1	0.4 ± 0.0
**Glass/HMDS**	6.9 ± 1.8	56.8 ± 0.5	27.6 ± 0.4	1.4 ± 0.2	7.4 ± 0.7	-
**Glass/P-SiO_2_/HMDS**	7.1 ± 0.6	60.2 ± 0.4	32.6 ± 0.2	0.0 ± 0.0	0.2 ± 0.0	-

It can be observed that the use of DMDClSi and HMDS silylating agents results in different C/Si ratios, with the glass/DMDClSi sample containing nearly twice as much carbon as the glass/HMDS and glass/P-SiO_2_/HMDS samples, which implies differences in the structure of the resulting surface layers. This difference is also reflected in the wettability of the sample surfaces (compare Tables 4 and 6). It was further observed that Na and Mg from the glass substrate were detectable in both DMDClSi- and HMDS-modified glass samples (glass/DMDClSi and glass/HMDS). In the HMDS-modified P-SiO_2_ sample (glass/P-SiO_2_/HMDS), however, the P-SiO_2_ coating, with a thickness of 127 ± 2 nm (Table 2), was sufficient to prevent the detection of substrate-derived Na and Mg (Table 6).

### 3.5 Abrasion Resistance of SiO_2_-based Coatings

To evaluate the abrasion resistance of the prepared coatings, glass/H-SiO_2_ and glass/P-SiO_2_/HMDS samples were subjected to a standardized abrasion test according to ISO 9211-4:2012.^48^ The test conditions were selected based on category C of the environmental durability classification, which corresponds to coatings intended for outdoor applications where high abrasion resistance is not a primary requirement. This category was chosen to provide a representative moderate-severity level for assessing the mechanical durability of the coatings.

Visual inspection was performed at regular intervals during the abrasion process (every 5 cycles, i.e., 10 rubbing strokes) to monitor the onset and progression of surface changes. Both coating types remained fully adhered to the substrate after 50 abrasion cycles (i.e., 100 rubbing strokes), with no visible delamination observed in either case. The glass/H-SiO_2_ sample showed the first signs of surface degradation after approximately 10 rubbing cycles, where minor scratch formation became visible on the coating surface. In contrast, the glass/P-SiO_2_/HMDS coating did not exhibit any detectable delamination or cracking of the thin film when inspected to the naked eye; however, a slight decrease in surface intensity (fading) was observed after approximately 10 cycles.

### 3.6 Effect of Soaking on Wettability and Optical Stability of the Samples

#### 3.6.1 Effect of Soaking on the Wettability of the Samples

The coating stability was investigated in greater detail by means of soaking experiments, in which the pH of the aqueous medium (X_1_), the soaking duration (X_2_), and the temperature of the solution (X_3_) were systematically varied. The experiments were carried out according to a three-factor, three-level Box–Behnken experimental design. Tables 7, 8, and 9 present the measured advancing (Θ_A_) and receding (Θ_R_) water contact angles, and the contact angle hysteresis (H) values determined after the soaking experiments for the glass/DMDClSi, glass/H-SiO_2_, and glass/P-SiO_2_/HMDS sample types, respectively. Using the Box–Behnken experimental design, the regression coefficients (β) were estimated from the measured responses by multiple regression analysis. The model coefficients for the advancing (Θ_A_) and receding (Θ_R_) contact angles are listed in Tables 10 and 11, respectively.

**Table 7:** Measured Advancing (Θ_A_) and Receding (Θ_R_) Water Contact Angles, the Derived Contact Angle Hysteresis (H), Equilibrium (Young) Contact Angle (θ_Young_), and Work of Adhesion (W_adh_) Values for the Glass/DMDClSi Sample after the Soaking Experiments (Prior to Soaking: Θ_A_ = 101 ± 1°, Θ_R_ = 100 ± 1°, H = 1 ± 1, Θ_Young_ = 101 ± 1°, W_adh_ = 60 ± 1 mN m^–1^)

**pH (X_1_)**	**t [h] (X_2_)**	**T [°C] (X_3_)**	**Θ_A_ [°]**	**Θ_R_ [°]**	**H [°]**	**Θ_Young_ [°]**	**W_adh_** **[mN m^–1^]**
2.0	2.0	42.4	98 ± 0	97 ± 0	1 ± 0	98 ± 0	63 ± 0
2.0	13.0	24.9	96 ± 3	94 ± 3	2 ± 0	95 ± 2	66 ± 3
2.0	13.0	59.9	91 ± 1	82 ± 1	9 ± 3	87 ± 1	77 ± 1
2.0	24.0	42.4	96 ± 4	95 ± 5	1 ± 0	96 ± 3	66 ± 4
7.0	2.0	24.9	102 ± 1	100 ± 2	2 ± 1	101 ± 1	59 ± 1
7.0	2.0	59.9	104 ± 1	99 ± 1	5 ± 1	102 ± 1	58 ± 1
7.0	13.0	42.4	102 ± 3	95 ± 2	7 ± 4	99 ± 2	62 ± 2
7.0	24.0	24.9	101 ± 1	87 ± 0	14 ± 1	94 ± 0	68 ± 1
7.0	24.0	59.9	99 ± 1	96 ± 1	3 ± 0	98 ± 1	63 ± 1
12.0	2.0	42.4	102 ± 0	99 ± 3	2 ± 3	101 ± 2	60 ± 2
12.0	13.0	24.9	102 ± 1	90 ± 3	11 ± 4	96 ± 2	65 ± 2
12.0	13.0	59.9	83 ± 1	74 ± 1	10 ± 1	79 ± 0	87 ± 1
12.0	24.0	42.4	86 ± 3	67 ± 0	19 ± 3	77 ± 2	90 ± 2

**Table 8:** Measured Advancing (Θ_A_) and Receding (Θ_R_) Water Contact Angles, the Derived Contact Angle Hysteresis (H), Equilibrium (Young) Contact Angle (θ_Young_), and Work of Adhesion (W_adh_) Values for the Glass/H-SiO_2_ Sample after the Soaking Experiments (Prior to Soaking: Θ_A_ = 95 ± 1°, Θ_R_ = 94 ± 1°, H = 1 ± 1°, Θ_Young_ = 95 ± 1°, W_adh_ = 67 ± 1 mN m^–1^)

**pH (X_1_)**	**t [h] (X_2_)**	**T [°C] (X_3_)**	**Θ_A_ [°]**	**Θ_R_ [°]**	**H [°]**	**Θ_Young_ [°]**	**W_adh_** **[mN m^–1^]**
2.0	2.0	42.4	74 ± 2	48 ± 2	25 ± 1	62 ± 1	107 ± 2
2.0	13.0	24.9	79 ± 4	67 ± 4	12 ± 0	73 ± 3	94 ± 3
2.0	13.0	59.9	72 ± 1	49 ± 1	23 ± 0	61 ± 1	108 ± 1
2.0	24.0	42.4	79 ± 0	69 ± 0	9 ± 1	74 ± 0	93 ± 0
7.0	2.0	24.9	87 ± 3	74 ± 5	13 ± 3	81 ± 3	85 ± 4
7.0	2.0	59.9	76 ± 0	58 ± 4	18 ± 4	67 ± 2	101 ± 2
7.0	13.0	42.4	85 ± 1	76 ± 1	9 ± 0	81 ± 1	85 ± 1
7.0	24.0	24.9	84 ± 0	67 ± 1	17 ± 0	76 ± 0	91 ± 1
7.0	24.0	59.9	66 ± 1	36 ± 1	29 ± 1	53 ± 1	117 ± 1
12.0	2.0	42.4	8 ± 1	7 ± 0	0 ± 0	8 ± 1	145 ± 0
12.0	13.0	24.9	50 ± 1	29 ± 4	21 ± 5	41 ± 2	128 ± 1
12.0	13.0	59.9	7 ± 0	5 ± 0	2 ± 0	6 ± 0	145 ± 0
12.0	24.0	42.4	15 ± 0	12 ± 0	4 ± 0	14 ± 0	144 ± 0

**Table 9:** Measured Advancing (Θ_A_) and Receding (Θ_R_) Water Contact Angles, the Derived Contact Angle Hysteresis (H), Equilibrium (Young) Contact Angle (θ_Young_), and Work of Adhesion (W_adh_) Values for the Glass/P-SiO_2_/HMDS Sample after the Soaking Experiments (Prior to Soaking: Θ_A_ = 93 ± 1°, Θ_R_ = 92 ± 1°, H = 1 ± 1°, Θ_Young_ = 93 ± 1°, W_adh_ = 70 ± 1 mN m^–1^)

**pH (X_1_)**	**t [h] (X_2_)**	**T [°C] (X_3_)**	**Θ_A_ [°]**	**Θ_R_ [°]**	**H [°]**	**Θ_Young_ [°]**	**W_adh_** [mN m–1]
2.0	2.0	42.4	68 ± 6	50 ± 2	18 ± 6	59 ± 3	110 ± 4
2.0	13.0	24.9	54 ± 0	35 ± 2	19 ± 2	45 ± 1	124 ± 1
2.0	13.0	59.9	57 ± 1	33 ± 5	24 ± 5	46 ± 2	123 ± 2
2.0	24.0	42.4	49 ± 1	27 ± 1	22 ± 1	39 ± 1	129 ± 1
7.0	2.0	24.9	87 ± 1	82 ± 2	5 ± 2	85 ± 1	80 ± 1
7.0	2.0	59.9	76 ± 1	62 ± 0	13 ± 1	69 ± 1	99 ± 1
7.0	13.0	42.4	76 ± 1	60 ± 1	16 ± 1	68 ± 1	100 ± 1
7.0	24.0	24.9	80 ± 0	71 ± 0	9 ± 0	76 ± 0	91 ± 0
7.0	24.0	59.9	62 ± 1	38 ± 0	24 ± 1	51 ± 1	119 ± 1
12.0	2.0	42.4	30 ± 1	16 ± 3	14 ± 3	24 ± 1	139 ± 1
12.0	13.0	24.9	25 ± 1	11 ± 1	15 ± 1	19 ± 1	142 ± 0
12.0	13.0	59.9	0 ± 0	0 ± 0	0 ± 0	0 ± 0	146 ± 0
12.0	24.0	42.4	33 ± 1	15 ± 0	18 ± 1	26 ± 1	139 ± 0

**Table 10:** Estimated Linear, Quadratic, and Interaction Regression Coefficients (β) for the Advancing Contact Angles (Θ_A_) Derived from the Box–Behnken Design for the Glass/DMDClSi, Glass/H-SiO_2_, and Glass/P-SiO_2_/HMDS Samples^a^

**Regression coefficient**	**Glass/DMDClSi**	**Glass/H-SiO_2_**	**Glass/P-SiO_2_/HMDS**
**β_0_**	96.52	58.84	51.84
**β_1_**	–0.78	–26.81	–17.57
**β_2_**	3.44	16.18	18.43
**β_3_**	–3.40	–1.09	–4.50
**β_11_**	–0.07	3.21	–2.71
**β_22_**	–2.81	–9.91	–6.43
**β_33_**	0.79	0.21	2.74
**β_12_**	–3.42	–1.75	5.70
**β_13_**	–2.91	–9.08	–7.04
***β*_23_**	–1.88	–1.88	–1.75

a β_0_ is the intercept; β_i_, β_ii_, and β_ij_ represent the linear, quadratic, and interaction effects of the coded variables, respectively.

**Table 11:** Estimated Linear, Quadratic, and Interaction Regression Coefficients (β) for the Receding Contact Angles (Θ_R_) Derived from the Box–Behnken Design for the Glass/DMDClSi, Glass/H-SiO_2_, and Glass/P-SiO_2_/HMDS Samples^a^

**Regression coefficient**	**Glass/DMDClSi**	**Glass/H-SiO_2_**	**Glass/P-SiO_2_/HMDS**
**β_0_**	89.53	43.92	36.64
**β_1_**	–5.34	–21.79	–12.97
**β_2_**	3.89	15.44	19.26
**β_3_**	–6.76	–1.08	–7.30
**β_11_**	–0.97	4.75	–2.63
**β_22_**	–3.15	–11.09	–8.24
**β_33_**	1.48	3.75	1.04
**β_12_**	–7.46	–5.40	5.43
**β_13_**	–2.46	–1.47	–2.05
**β_23_**	1.49	–3.69	–3.45

a β_0_ is the intercept; β_i_, β_ii_, and β_ij_ represent the linear, quadratic, and interaction effects of the coded variables, respectively.

The evaluation of the experimental soaking data (Tables 7–9) and the Box–Behnken regression model (Tables 10 and 11) clearly identifies pH (X_1_) as the dominant factor determining the stability of all investigated coatings. Under neutral conditions (pH 7), all surfaces retained high advancing contact angles (Θ_A_ ≈ 101–104° for glass/DMDClSi, 84–87° for glass/H-SiO_2_, and 76–87° for glass/P-SiO_2_/HMDS), whereas acidic media caused moderate deterioration, and alkaline environments (pH 12) led to severe loss of hydrophobicity. In particular, complete wetting was observed for the silica-based coatings at pH 12 and 59.9 °C (Θ_A_ ≈ 7° for glass/H-SiO_2_ and 0° for glass/P-SiO_2_/HMDS), indicating extensive surface degradation. (Tables 8 and 9)

As discussed in our previous work,^18^ the decrease in water contact angles for silica-based coatings can be attributed to two main mechanisms. Firstly, water molecules penetrate the boundary layer of the solid matrix during soaking, slightly reducing the hydrophobic character of the surface. Secondly, in strongly acidic or alkaline media, hydrolysis of the siloxane bridges generates polar silanol groups, which orient toward the polar water molecules, thereby increasing the surface hydrophilicity. This effect is particularly pronounced for glass/H-SiO_2_ and glass/P-SiO_2_/HMDS, leading to a significant decrease in contact angle values. In the case of the glass/DMDClSi sample, only a thin layer of surface-bound Si–O–Si linkages is formed during silylation, without the development of an extended siloxane network. Consequently, hydrolysis plays only a minor role in the reduction of the contact angle, and the slight decrease observed is mainly attributable to the penetration of water into the boundary layer and to limited chemical heterogeneity within the ultrathin surface film.

These trends are quantitatively confirmed by the regression coefficients (Tables 10 and 11). The strongly negative linear pH terms (β_1_) for both Θ_A_ and Θ_R_ confirm that increasing alkalinity is the primary driver of wettability loss. This effect is especially pronounced for glass/H-SiO_2_ and glass/P-SiO_2_/HMDS, while glass/DMDClSi shows only weak pH sensitivity, consistent with its higher chemical resistance. The positive intercepts (β_0_) follow the same ranking as the initial hydrophobicity of the samples, further supporting the experimental observations. Temperature (X_3_) acts as a secondary but significant destabilizing factor. Experimentally, higher temperatures reduced both Θ_A_ and Θ_R_ and increased contact angle hysteresis (Tables 7–9). In the regression model, this is reflected by negative linear temperature coefficients (β_3_) and, in some cases, positive quadratic terms (β_33_), indicating nonlinear thermal effects. The interaction between pH and temperature (β_13_) is strongly negative for the silica-based coatings, demonstrating that alkaline and thermal stresses act synergistically to accelerate surface degradation. Soaking time (X_2_) alone has a weaker and nonmonotonic influence. While the linear time terms (β_2_) are slightly positive near the design center, the negative quadratic coefficients (β_22_) indicate curvature and progressive degradation at longer exposure times. This agrees with the experimental finding that prolonged soaking becomes critical mainly under extreme pH or high-temperature conditions. Correspondingly, the interaction terms β_12_ (pH–time) and β_23_ (time–temperature) show that extended soaking time intensifies the destabilizing effects of chemical and thermal stress, particularly for glass/H-SiO_2_ and glass/P-SiO_2_/HMDS sample types (Tables 8 and 9). Receding contact angles (Θ_R_) exhibit even stronger sensitivities than advancing contact angles, reflecting the high responsiveness of Θ_R_ to surface heterogeneity and damage. Both experimental data and regression coefficients show that Θ_R_ decreases more rapidly than Θ_A_ under alkaline and thermal stress, and this is accompanied by a marked increase in CA hysteresis. Under extreme conditions, glass/H-SiO_2_ and glass/P-SiO_2_/HMDS samples develop pronounced CA hysteresis (up to 20–30°), whereas glass/DMDClSi preserves consistently low hysteresis, indicating a more homogeneous and stable surface, whereas the high hysteresis of the silica-based coatings suggests that the surface has undergone partial chemical and morphological degradation (Tables 7–9).

The changes observed in the equilibrium (Young) contact angle and the corresponding work of adhesion (W_adh_) further support the conclusions drawn from the advancing and receding contact angle measurements (Tables 7–9). For all investigated samples, a decrease in Θ_Young_ was accompanied by a systematic increase in W_adh_, consistent with the Young–Dupré relationship. The glass/DMDClSi surface exhibited only moderate changes across the experimental conditions. Its Young contact angle remained relatively high (77–102°), while the work of adhesion increased only from the initial value of 60 mN m^–1^ to a maximum of approximately 90 mN m^–1^ under the most aggressive soaking conditions (Table 7). These results confirm the high chemical stability of the DMDClSi-modified surface and indicate that only limited changes occurred in the surface chemistry during soaking. In contrast, the silica-based coatings showed a much stronger response to the soaking conditions. For the glass/H-SiO_2_ sample, Θ_Young_ decreased from the initial value of 95° to as low as 6–14° under alkaline conditions, accompanied by a substantial increase in W_adh_ to 144–145 mN m^–1^ (Table 8). Similarly, the glass/P-SiO_2_/HMDS coating exhibited a decrease in Θ_Young_ from 93° to 0–26°, while the adhesion work increased from 70 mN m^–1^ to 139–146 mN m^–1^ (Table 9). The nearly complete disappearance of the Young contact angle and the simultaneous increase of the work of adhesion toward the theoretical limit predicted by the Young–Dupré relationship indicate the development of highly hydrophilic surfaces and, in the most severe cases, complete wetting.

The dependence of Θ_Young_ and W_adh_ on pH, temperature, and soaking duration follows the same trends observed for the advancing and receding contact angles. Neutral conditions generally preserved relatively high Θ_Young_ and low W_adh_ values, whereas acidic media caused moderate changes and alkaline environments led to the most pronounced deterioration. Elevated temperatures further accelerated these effects, particularly for the silica-containing samples. The strong increase in adhesion work under alkaline and thermal stress indicates the formation of polar surface functionalities, most likely silanol groups generated by hydrolysis of siloxane bonds, which enhance water–surface interactions. Consequently, the evolution of Θ_Young_ and W_adh_ provides additional quantitative evidence that the silica-based coatings undergo substantial chemical degradation under aggressive soaking conditions, while the DMDClSi-modified glass retains a significantly higher level of hydrophobicity and interfacial stability.

Overall, the combined experimental–model analysis demonstrates that pH is the primary driver of wettability loss, temperature is the main accelerating factor, and soaking time modulates these effects through nonlinear and interaction effects. The most severe degradation occurs when all three variables reach their extreme levels simultaneously, particularly for silica-based coatings, while the DMDClSi-modified glass surface exhibits the highest resistance across the investigated design space. Although small deviations are observed under extreme conditions, the model reliably reflects the overall trends, quantitatively representing the influence of pH, soaking time, and temperature on contact angle evolution and surface stability.

#### 3.6.2 Effect of Soaking on the Light Transmittance of the Samples

Tables 12 and 13 present the maximum transmittance (T_max_) and the average transmittance increase (ATI) values for the glass/H-SiO_2_ and glass/P-SiO_2_/HMDS samples, respectively, after soaking, as measured by UV–Vis spectrophotometry. The experimental data were modeled using a Box–Behnken design with a quadratic polynomial model, enabling the estimation of the β coefficients that quantify the effects of pH, soaking time, and temperature on T_max_ and ATI. The resulting coefficients are presented in Table 14.

**Table 12:** Maximum Transmittance (T_max_) and the Average Transmittance Increase (ATI) Values of the Glass/H-SiO_2_ Samples after the Soaking Experiments Obtained from UV–Vis Spectrophotometry (Prior to Soaking: T_max_ = 99.60 ± 0.07%, ATI = 6.5 ± 0.1%)

**pH (X_1_)**	**t [h] (X_2_)**	**T [°C] (X_3_)**	**T_max_ [%]**	**ATI [%]**
2.0	2.0	42.4	99.6 ± 0.1	5.8 ± 0.1
2.0	13.0	24.9	99.4 ± 0.2	5.7 ± 0.1
2.0	13.0	59.9	99.4 ± 0.0	5.4 ± 0.4
2.0	24.0	42.4	99.4 ± 0.0	5.3 ± 0.1
7.0	2.0	24.9	99.6 ± 0.2	5.9 ± 0.1
7.0	2.0	59.9	99.4 ± 0.0	5.5 ± 0.2
7.0	13.0	42.4	98.9 ± 0.1	5.0 ± 0.1
7.0	24.0	24.9	98.4 ± 1.6	5.0 ± 1.3
7.0	24.0	59.9	99.7 ± 0.0	6.0 ± 0.1
12.0	2.0	42.4	93.2 ± 2.1	0.4 ± 1.7
12.0	13.0	24.9	99.5 ± 0.1	5.6 ± 0.1
12.0	13.0	59.9	92.0 ± 0.1	0.1 ± 0.1
12.0	24.0	42.4	93.2 ± 0.6	0.8 ± 0.4

**Table 13:** Maximum Transmittance (T_max_) and the Average Transmittance Increase (ATI) Values of the Glass/P-SiO_2_/HMDS after the Soaking Experiments Obtained from UV–Vis Spectrophotometry (Prior to Soaking: T_max_ = 99.68 ± 0.05%, ATI = 6.0 ± 0.2%)^a^

**pH (X_1_)**	**t [h] (X_2_)**	**T [°C] (X_3_)**	**T_max_ [%]**	**ATI [%]**
2.0	2.0	42.4	99.6 ± 0.1	6.1 ± 0.1
2.0	13.0	24.9	99.6 ± 0.0	6.1± 0.1
2.0	13.0	59.9	99.6 ± 0.0	6.0 ± 0.1
2.0	24.0	42.4	99.6 ± 0.0	6.1 ± 0.0
7.0	2.0	24.9	99.6 ± 0.2	6.0 ± 0.1
7.0	2.0	59.9	99.6 ± 0.0	6.1 ± 0.0
7.0	13.0	42.4	99.5 ± 0.0	5.7 ± 0.0
7.0	24.0	24.9	99.6 ± 0.0	5.9 ± 0.1
7.0	24.0	59.9	99.4 ± 0.0	4.9 ± 0.2
12.0	2.0	42.4	99.6 ± 0.0	6.1 ± 0.2
12.0	13.0	24.9	99.5 ± 0.0	5.7 ± 0.0
12.0	24.0	42.4	99.1 ±0.2	6.2 ± 0.2
*12.0	13.0	59.9	-	-

a Optical parameters could not be determined due to the severe degradation/loss of transparency of the coating under these extreme alkaline–thermal conditions.

**Table 14:** Estimated Linear, Quadratic, and Interaction Regression Coefficients (β) for the Maximum Transmittance (T_max_) and Average Transmittace Increase (ATI) Derived from the Box–Behnken Design for the Glass/H-SiO_2_ and Glass/P-SiO_2_/HMDS Samples^a^

**Regression coefficient**	**T_max_** **(Glass/H-SiO_2)_**	**T**_**max**_ **(Glass/P-SiO_2_/HMDS)**	**ATI** **(Glass/H-SiO_2_)**	**ATI** **(Glass/P-SiO_2_/HMDS)**
**β_0_**	97.73	99.50	4.29	5.91
**β_1_**	–2.49	–0.12	–1.91	–0.08
**β_2_**	–0.14	–0.09	–0.06	–0.15
**β_3_**	–0.80	–0.06	–0.65	–0.13
**β_11_**	1.06	0.02	0.83	–0.14
**β_22_**	0.21	–0.01	0.13	–0.07
**β_33_**	–0.40	–0.02	–0.43	0.06
**β_12_**	0.05	–0.13	0.22	0.03
**β_13_**	–1.88	–0.06	–1.30	0.03
**β_23_**	0.38	–0.05	0.35	–0.28

a β_0_ is the intercept; β_i_, β_ii_, and β_ij_ represent the linear, quadratic, and interaction effects of the coded variables , respectively.

UV–Vis spectrophotometry reveals fundamentally different stability profiles for glass/H-SiO_2_ and glass/P-SiO_2_/HMDS coatings under combined chemical and thermal stress. Both samples maintain high transparency under acidic and neutral conditions (pH 2 and pH 7; T_max_ ≈ 99–100%, ATI ≈ 5–6%). Minor reductions in T_max_ and ATI are observed at longer soaking times or elevated temperatures, indicating a slight, time- and temperature-dependent optical response, particularly for glass/H-SiO_2_. Under alkaline conditions (pH 12), the two coatings demonstrate markedly different optical stability behavior. Glass/H-SiO_2_ undergoes severe degradation, with T_max_ dropping to ∼92–93% and ATI decreasing to ∼0–1% at elevated temperature and extended soaking time, highlighting strong synergistic effects of time and temperature. In contrast, glass/P-SiO_2_/HMDS remains essentially unaffected across the entire design space, with T_max_ ≥ 99.1% and ATI ≈ 5.7–6.2%, demonstrating negligible sensitivity to both prolonged soaking and thermal stress (Tables 12 and 13). These results demonstrate that the glass/P-SiO_2_/HMDS coating exhibits outstanding optical robustness, with minimal dependence on pH, soaking time, and temperature within the investigated range. In terms of wettability, glass/H-SiO_2_ and glass/P-SiO_2_/HMDS behave similarly under the soaking conditions. The difference in optical response arises from the HMDS-derived functional layer: although its degradation slightly reduces the water-repellent properties, it does not significantly affect the transmittance, as even the unmodified glass/P-SiO_2_ coating already exhibits high optical transparency.^36,37^

The regression coefficients obtained from the Box–Behnken model are fully consistent with the trends observed directly in the maximum transmittance and average transmittance increase data (compare Tables 12, 13, and 14). For the glass/H-SiO_2_ coatings, the strong negative linear effects of pH (β_1_ = −2.49 for T_max_ and −1.91 for ATI) and temperature (β_3_ = −0.80 for T_max_ and −0.65 for ATI) quantitatively confirm that increasing pH and elevated temperature are the dominant drivers of optical degradation (Table 14). This is in excellent agreement with the measured data where T_max_ and ATI collapse at pH 12, particularly at 59.9 °C (Table 12). The pronounced negative interaction between pH and temperature (β_13_ = −1.88 for T_max_ and −1.30 for ATI) further supports the experimentally observed synergistic effect: under combined alkaline and thermal stress, glass/H-SiO_2_ rapidly loses transparency and optical functionality. The positive quadratic terms in pH (β_11_ > 0) indicate a pronounced nonlinear pH effect, consistent with the sharp transition from stable behavior at pH 2–7 to severe degradation at pH 12. In contrast, the glass/P-SiO_2_/HMDS samples are characterized by uniformly small coefficients across all linear, quadratic, and interaction terms. This directly reflects the raw UV–Vis results, which show T_max_ ≥ 99% and ATI ≈ 5.7–6.2% over the entire design space (compare Tables 13 and 14). The negligible β-values indicate that neither pH, soaking time, nor temperature significantly influences the optical performance of the glass/P-SiO_2_/HMDS sample surface. Thus, the regression analysis results align with the measured data, demonstrating that glass/P-SiO_2_/HMDS sample type exhibits high optical resistance under simultaneous alkaline and thermal conditions, whereas the glass/H-SiO_2_ sample undergoes a significant loss of optical performance. While minor differences appear under extreme conditions, the quadratic model effectively represents the main experimental patterns, quantitatively describing the effects of pH, soaking time, and temperature on the transmittance of the coatings.

### 3.7 FE-SEM Investigation of the As-Prepared and Soaked Samples

Owing to the instrumental requirements of FE-SEM characterization, the measurements were performed on coatings deposited onto silicon substrates instead of glass substrates enabling reliable imaging and accurate evaluation of the samples. Figure 3 shows representative cross-sectional FE-SEM images taken from the Si/H-SiO_2_ (a) and Si/P-SiO_2_/HMDS (b) sample types. The coating thickness values were determined from cross-sectional FE-SEM images (Figure 3) using ImageJ software. The thicknesses of the Si/H-SiO_2_ and Si/P-SiO_2_/HMDS coatings were determined to be 120 ± 6 nm and 121± 11 nm, respectively. These values are in good agreement with the thickness values obtained from the analysis of the UV–Vis transmittance spectra and ellipsometric porosimetry data of the samples on glass substrates (Table 2).

**Figure 3:**
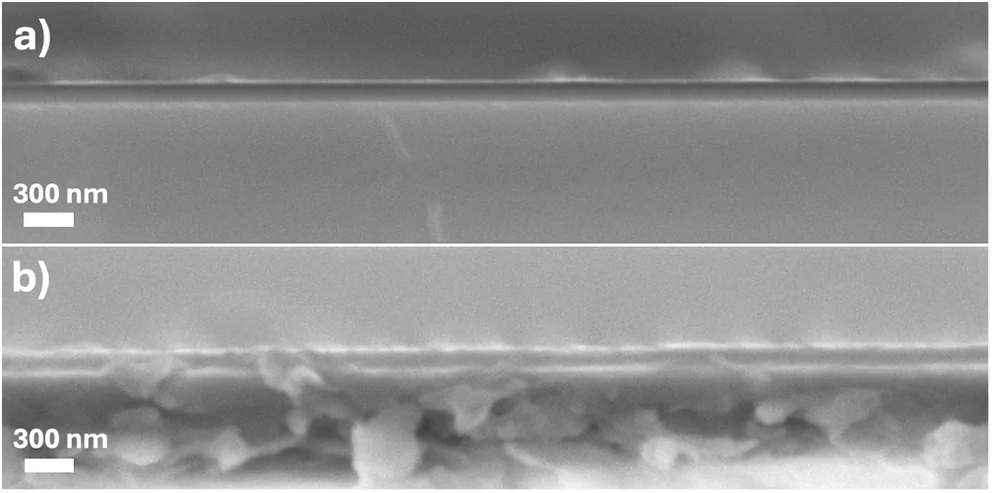
Cross-sectional FE-SEM images of the hybrid silica (Si/H-SiO_2_) (a) and hexamethyldisilazane-modified silica (Si/P-SiO_2_/HMDS) (b) coatings on silicon substrates.

The surface morphology of the as-prepared and soaked (pH 12, 59.9 °C,13 h) Si/DMDClSi, Si/H-SiO_2_, and Si/P-SiO_2_/HMDS samples was investigated by top-view FE-SEM imaging (Figure 4). All three as-prepared sample types exhibited highly homogeneous surface morphologies. After soaking at pH 12 and 59.9 °C for 13 h, surface inhomogeneities emerged in all cases, although their extent and morphological characteristics varied significantly among the different sample types (Figure 4). It should be noted that some of the localized surface features visible on the soaked samples can be attributed to drying-mark residues formed during sample preparation after the soaking treatment. Consequently, these features should be distinguished from the more general morphological changes associated with coating degradation. Despite these drying-related features, the FE-SEM images clearly reveal that exposure to strongly alkaline conditions resulted in measurable modifications of the surface morphology. The morphological changes observed by FE-SEM are in qualitative agreement with the wettability results obtained for the corresponding samples on glass substrates (Tables 7–9).

**Figure 4:**
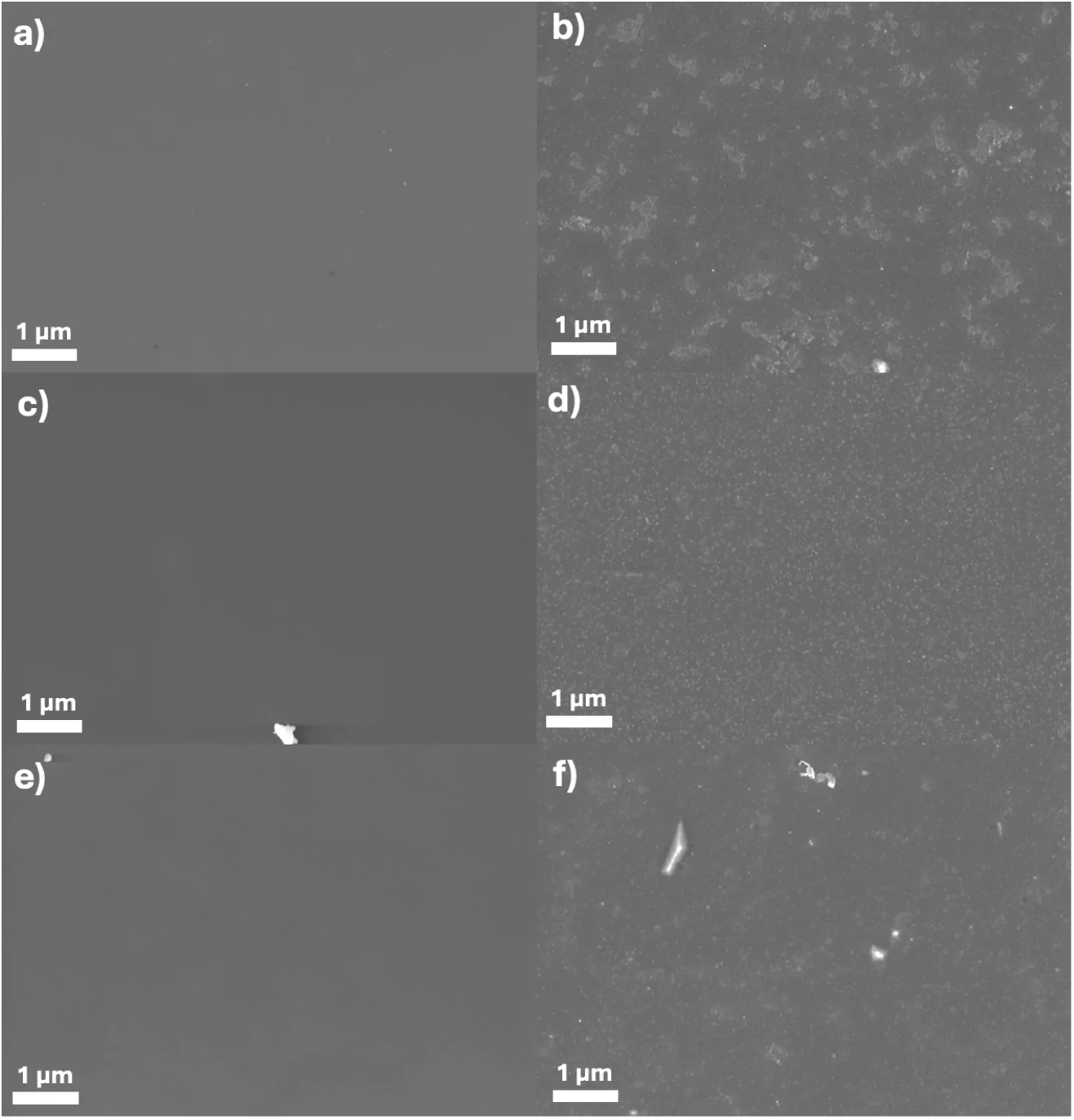
Top-view FE-SEM images of the as-prepared (a, c, e) and soaked (pH 12, 59.9 °C,13 h) (b, d, f) Si/DMDClSi (a, b), Si/H-SiO_2_ (c, d), and Si/P-SiO_2_/HMDS (e, f) samples.

### 3.8 Surface Topography and Roughness Analysis by AFM

Representative AFM images of the as-prepared and soaked surfaces are shown in Figure 5. The images provide qualitative information on the morphological changes induced by exposure to the most severe soaking conditions (pH 12, 59.9 °C, 13 h). Quantitative surface characterization derived from the AFM height images was performed through evaluation of the root mean square roughness (Sq) and arithmetic mean roughness (Sa), as summarized in Table 15. For the soaked samples, drying-mark features formed during sample preparation were flattened during image processing to improve visualization of the underlying surface morphology and to ensure a more accurate assessment of the actual topographical changes induced by the soaking treatment. Prior to soaking, all investigated surfaces exhibited low roughness values, indicating smooth and homogeneous coatings. Among the as-prepared samples, the glass/H-SiO_2_ coating showed the lowest (Sq = 0.27 nm, Sa = 0.20 nm), while the glass/P-SiO_2_/HMDS coating exhibited the highest (Sq = 3.21 nm, Sa = 2.55 nm) roughness. The glass/DMDClSi sample showed intermediate roughness values (Sq = 2.33 nm, Sa = 1.17 nm). After exposure to the most severe soaking conditions, a marked increase in roughness was observed for all samples when the entire scanned area was considered. The increase was most pronounced for glass/DMDClSi and glass/P-SiO_2_/HMDS, with Sq values rising to 101.1 and 94.8 nm, respectively, while glass/H-SiO_2_ showed a more moderate increase (Sq = 34.8 nm). These elevated values are primarily attributed to localized drying marks formed during sample preparation, which significantly contribute to the apparent roughness. When these regions were excluded, the calculated roughness values decreased substantially for all samples. For the glass/H-SiO_2_ sample, the corrected roughness values (Sq = 1.70 nm, Sa = 0.55 nm) remained only moderately higher than those of the as-prepared surface. Similarly, the corrected roughness values of the glass/DMDClSi (Sq = 13.9 nm, Sa = 9.0 nm) and glass/P-SiO_2_/HMDS (Sq = 8.3 nm, Sa = 5.2 nm) samples were markedly reduced compared with the uncorrected data. Nevertheless, all corrected roughness values remained higher than those measured for the corresponding as-prepared samples, demonstrating that morphological changes occurred during soaking under strongly alkaline conditions. The AFM results are in good agreement with the FE-SEM observations (Figure 4). While the as-prepared samples exhibited homogeneous surface morphologies, the soaked samples showed distinct surface inhomogeneities and the appearance of localized surface features. Both techniques therefore confirm that exposure to highly alkaline media at elevated temperature induces measurable morphological changes in all investigated systems. A comparison between the corrected AFM roughness values (excluding drying-mark-related features, Table 15) and the UV–Vis transmittance of the as-prepared and soaked samples (Tables 12 and 13) indicates that surface morphological changes are accompanied by a strong deterioration of optical performance under alkaline–thermal conditions. For the glass/H-SiO_2_ coating, an increase in roughness is associated with a measurable decrease in both T_max_ and ATI, reflecting partial optical degradation. In contrast, the glass/P-SiO_2_/HMDS sample undergoes a much more severe degradation (Tables 12, 13, and 15). This indicates that, while both systems exhibit morphological changes, the extent of optical degradation is markedly different, suggesting distinct degradation pathways rather than a simple one-to-one correlation between roughness and transmittance. Importantly, the extent of roughness increase does not directly correlate with the observed deterioration of the wetting properties. For example, the glass/H-SiO_2_ sample exhibited only a relatively small increase in corrected roughness, yet showed a dramatic decrease in Young’s contact angle and a substantial increase in the work of adhesion after soaking. In contrast, the glass/DMDClSi sample retained comparatively high contact angle values despite exhibiting the largest increase in corrected roughness (Tables 7 and 15). These observations indicate that the loss of hydrophobicity cannot be explained solely by morphological changes. Instead, the wettability results suggest that alterations in surface chemistry play the dominant role in the degradation process. In particular, hydrolysis of siloxane bonds and the consequent formation of hydrophilic silanol groups under strongly alkaline conditions are likely responsible for the pronounced decrease in Young’s contact angle and the corresponding increase in the work of adhesion observed for the silica-based coatings (Tables 8 and 9). Thus, the FE-SEM and AFM analyses provide complementary evidence of morphological degradation, whereas the wettability measurements demonstrate that chemical modification of the surface is the primary factor determining the deterioration of hydrophobic performance.

**Figure 5:**
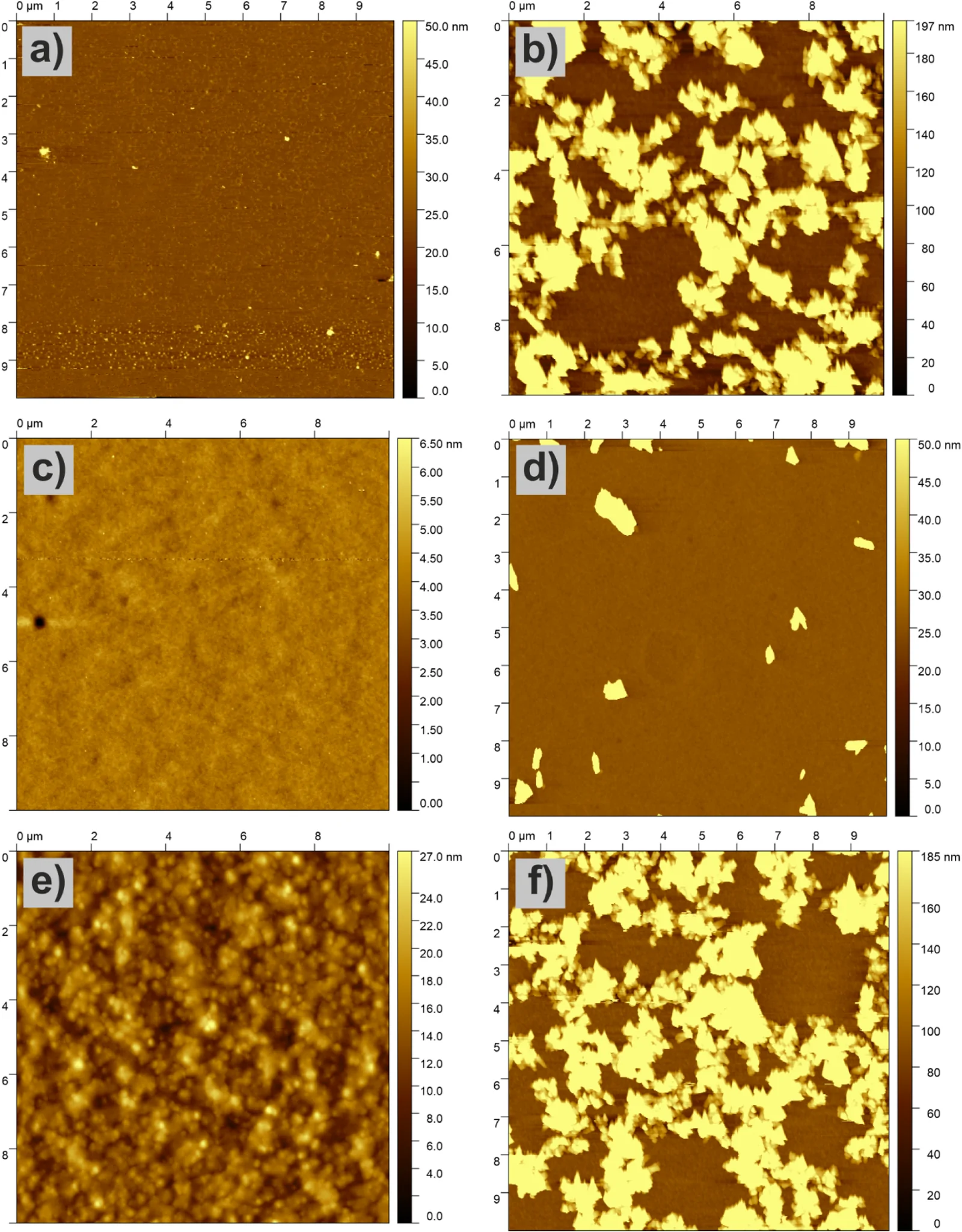
Top-view AFM images of the as-prepared (a, c, e) and soaked (pH 12, 59.9 °C,13 h) (b, d, f) glass/DMDClSi (a, b), glass/H-SiO_2_ (c, d), and glass/P-SiO_2_/HMDS (e, f) samples. For the soaked samples, drying-mark features were flattened during image processing to facilitate visualization of the underlying surface morphology.

**Table 15:** Root Mean Square (RMS) Roughness (Sq) and Mean Roughness (Sa) Values Calculated for the As-Prepared and Soaked (pH 12, 59.9 °C,13 h) Surfaces of the Samples Shown in Figure 5 ^a^

						**Sq [nm]**	**Sa [nm]**
**Sample**	**Sq [nm]**	**Sa [nm]**	**Sample**	**Sq [nm]**	**Sa [nm]**	**Excluding drying marks**	
**Glass/DMDClSi (as prepared)**	2.33	1.17	**Glass/DMDClSi** **(soaked)**	101.1	82.1	13.9	9.0
**Glass**/**H-SiO**_2_(as prepared)	0.27	0.2	**Glass**/**H-SiO_2_** **(soaked)**	34.8	9.8	1.70	0.55
**Glass**/**P-SiO**_**2**_/**HMDS** **(as prepared)**	3.21	2.55	**Glass**/**P-SiO**_**2**_/**HMDS** **(soaked)**	94.8	77.3	8.3	5.2

a For the soaked samples, these values were calculated over the entire area and also excluding the drying marks.

### 3.9 ATR-FTIR Investigation of the As-Prepared and Soaked Glass/H-SiO_2_ Samples

ATR-FTIR spectroscopy was employed to investigate chemical changes in the glass/H-SiO_2_ coatings induced by alkaline soaking at pH 12 and 59.9 °C for 13 h. The spectrum of the bare glass substrate, used as a reference, is characterized by broad absorption bands centered at approximately 908 cm^–1^ and 770 cm^–1^, assigned to Si–O–Si vibrations of the silicate network (Figure 6). Similar features are present in the coated samples, indicating a significant contribution of the glass substrate to the overall ATR-FTIR response. In the as-prepared glass/H-SiO_2_ sample, a distinct absorption band at 1277 cm^–1^ together with a weak band at ∼2980 cm^–1^ is observed, which can be attributed to Si–CH_3_ groups originating from the organic components of the hybrid silica network (Figure 6). After alkaline soaking, these characteristic bands are no longer detectable (Figure 6), indicating the degradation of methyl functionalities under strongly basic conditions. This chemical transformation is consistent with the pronounced decrease in advancing and receding water contact angles from 95° and 94° to 7° and 5°, respectively (Table 8), confirming a substantial loss of surface hydrophobicity. No additional absorption bands attributable to Si–OH groups or molecular water can be clearly resolved in either the as-prepared or soaked samples. In particular, no discernible O–H stretching band (3200–3600 cm^–1^), H–O–H bending vibration (∼1630 cm^–1^), or Si–OH-related feature near ∼950 cm^–1^ is observed. This suggests that such species are either present in low concentrations below the detection limit of ATR-FTIR or are spectroscopically masked by the dominant silicate glass background. Importantly, the chemical modifications identified by FTIR provide direct evidence for the mechanism underlying the wetting behavior discussed in Subsection 3.8. While AFM analysis indicated that changes in surface roughness do not correlate with the observed wettability degradation, the FTIR results demonstrate that the loss of hydrophobicity is primarily driven by chemical alterations of the surface, namely the degradation of Si–CH_3_ groups under alkaline conditions (soaking at pH 12 and 59.9 °C for 13 h).

**Figure 6:**
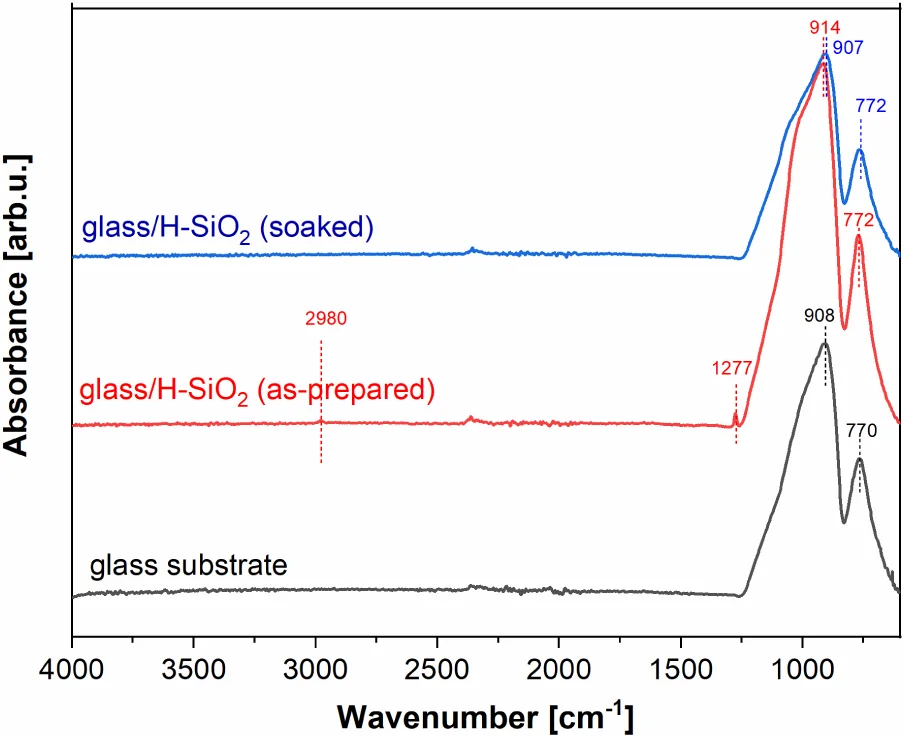
ATR-FTIR spectra of the bare glass substrate and hybrid silica coatings deposited on glass substrates (glass/H-SiO_2_), shown in the as-prepared state and after soaking (pH 12, 59.9 °C, 13 h).

## 4 Conclusions

This work presents a comprehensive comparative study of three distinct methodologies for hydrophobizing glass substrates: direct silanization (glass/DMDClSi), and the application of two functional coatings—hybrid silica (glass/H-SiO_2_) and hexamethyldisilazane-modified silica (glass/P-SiO_2_/HMDS). All surface treatments yielded hydrophobic surfaces, with advancing water contact angles exceeding 90° and very low contact angle hysteresis (∼1°), confirming chemically and morphologically homogeneous water-repellent surfaces. Surface free energy analysis further corroborated the hydrophobic character of the investigated systems, with the lowest values observed for the DMDClSi-modified surface and a clear dominance of the dispersive component over the polar component, consistent with the contact angle trends and the presence of nonpolar surface functionalities. In addition, both silica-based coatings (glass/H-SiO_2_ and glass/P-SiO_2_/HMDS) significantly enhanced the light transmission of the glass substrates, achieving maximum transmittance values above 99%, together with well-defined porosity (34–35%) and uniform layer thicknesses of 127 nm. Ellipsometric porosimetry revealed clear structural differences between the silica-based coatings. The glass/H-SiO_2_ coating exhibited a relatively homogeneous mesoporous network with a narrow pore size distribution centered at ∼2 nm and only minor hysteresis, whereas the glass/P-SiO_2_/HMDS coating displayed a type IV isotherm with a pronounced H2-type hysteresis loop, indicating a more heterogeneous interconnected mesoporous structure with larger pores, pore constrictions, and pore-network effects. These EP-derived differences were reflected in the determined optical and structural parameters. The HMDS-modified coating showed a lower refractive index (n = 1.225 vs 1.259), higher total and open porosity (48% vs 38% and 43% vs 37%), but a lower specific surface area (245 vs 1124 m^2^ /cm^3^), confirming larger, less surface-dense pores compared to the compact high-surface-area H-SiO_2_ network. The durability of the hydrophobic functionality and optical properties was systematically evaluated under combined chemical and thermal stress through soaking experiments designed according to a three-factor, three-level Box–Behnken framework. The results identified pH as the dominant degradation parameter, while temperature and exposure time acted as synergistic accelerators. Under strongly alkaline–thermal conditions, loss of Si–CH_3_ functionalities was identified as the primary mechanism controlling hydrophobic failure of the glass/H-SiO_2_ sample, as confirmed by ATR-FTIR analysis. This chemical transformation, rather than morphological roughening, was shown to be the key factor controlling wettability loss. The evolution of light transmittance under soaking conditions further clarified the degradation behavior. The glass/H-SiO_2_ coating exhibited pronounced optical deterioration across a broad range of alkaline–thermal conditions, with reduced maximum transmittance (T_max_ ≈ 92–93%) and negligibleaverage transmittance increase values (ATI ≈ 0–1%), highlighting strong synergistic effects of pH, temperature, and exposure time. In contrast, the glass/P-SiO_2_/HMDS system showed excellent optical stability across most of the investigated conditions, preserving high transparency (T_max_ ≥ 99.1%; ATI ≈ 5.7–6.2%) with minimal sensitivity to environmental variables, with degradation occurring only under the most severe alkaline–thermal conditions (pH 12, 13 h, 59.9 °C). Wettability stability tests further revealed a clear performance hierarchy among the investigated coatings. The glass/DMDClSi surface exhibited the highest resistance to wetting degradation across the entire experimental domain, maintaining relatively high advancing contact angles and low hysteresis even under strongly alkaline and elevated temperature conditions,showing substantially greater resistance to wetting degradation than both silica-based coatings. Among the silica-based systems, glass/P-SiO_2_/HMDS showed markedly improved hydrophobic stability compared to glass/H-SiO_2_, retaining water-repellent properties under most investigated conditions and undergoing severe wetting loss only under the most extreme alkaline–thermal exposure. The glass/H-SiO_2_ system exhibited the lowest wetting stability, including complete wetting under the most severe conditions. When optical performance and wettability are evaluated simultaneously, a clear functional trade-off emerges. The glass/DMDClSi coating provides superior wetting resistance but lacks the porosity-driven optical enhancement. The glass/H-SiO_2_ coating offers high surface area but suffers from poor stability in both optical and wetting properties under environmental stress. In contrast, the glass/P-SiO_2_/HMDS system represents the best overall compromise, maintaining high transparency (>99%) and beneficial optical properties under most conditions while preserving acceptable hydrophobic performance, except under extreme alkaline–thermal exposure. Importantly, the observed functional behavior can be directly correlated with the EP-derived pore structure and the ATR-FTIR results. The dense, high-surface-area network of glass/H-SiO_2_ is more susceptible to hydrolysis-induced loss of Si–CH_3_ groups and subsequent silanol formation, leading to rapid degradation of hydrophobicity. In contrast, the more open mesoporous structure of glass/P-SiO_2_/HMDS contributes to improved retention of both optical transparency and water repellency under most investigated conditions.

Overall, the combined structural, chemical, optical, and mechanical analyses demonstrate that no single coating optimizes all properties simultaneously. However, the glass/P-SiO_2_/HMDS coating delivers the most balanced performance among the investigated systems by combining high transparency, porosity-driven optical enhancement, and comparatively robust hydrophobic stability. Although all coatings eventually degrade under extreme alkaline–thermal conditions, the HMDS-modified system significantly extends functional stability relative to glass/H-SiO_2_, while glass/DMDClSi provides the benchmark for maximum wetting resistance. These results highlight the critical role of surface chemistry and pore structure in developing multifunctional coatings where optical performance and interfacial stability must be simultaneously optimized. The combination of excellent optical transparency (T_max_ > 99%), low contact angle hysteresis, and enhanced resistance to environmental degradation makes the glass/P-SiO_2_/HMDS coating particularly attractive for applications requiring both optical clarity and long-term water repellency. Potential application areas include self-cleaning architectural and automotive glazing, protective cover glasses for electronic displays, optical sensors, photovoltaic cover plates, and antifouling transparent surfaces exposed to outdoor environments.
